# Systematic review of protective factors related to academic resilience in children and adolescents: unpacking the interplay of operationalization, data, and research method

**DOI:** 10.3389/fpsyg.2024.1405786

**Published:** 2024-08-21

**Authors:** Wangqiong Ye, Nani Teig, Sigrid Blömeke

**Affiliations:** ^1^Faculty of Educational Sciences, Centre for Educational Measurement, University of Oslo, Oslo, Norway; ^2^Department of Teacher Education and School Research, Faculty of Educational Sciences, University of Oslo, Oslo, Norway

**Keywords:** academic resilience, disadvantaged students, protective factors, operationalization, large-scale assessment

## Abstract

Identifying protective factors that promote academic resilience is vital. Nevertheless, due to the variations in the operationalizations of academic resilience, timeframes, data sources, and employed research methods, it remains unclear whether the impact of protective factors identified across studies can be attributed to the factors themselves or to these variations. By addressing these uncertainties, this study aims to provide an overview of the protective factors that have been extensively investigated in academic resilience and their degree of influence. A literature search found 119 empirical studies on protective factors in education settings for children and adolescents. The review analyzed five protective factors groups (individual, family, school, peer, community), three operationalizations of academic resilience (simultaneous, progressive, instrumental), two timeframes (longitudinal, non-longitudinal), three data sources (self-collected, national/local assessments, international large-scale assessments), and commonly employed research methods. The studies analyzed in this review yielded mixed results regarding the impact of the examined protective factors, with measurement instruments and statistical power playing a significant role in explaining the variations. Individual and school-level characteristics emerged as the most well-studied protective factors; individual characteristics were often investigated through “instrumental” operationalization and structural equational models, whereas school-level characteristics were typically explored through “simultaneous” or “progressive” operationalizations and multilevel modeling. Approximately 31 and 16% of the studies utilized national assessments and international large-scale assessment data, respectively. Both data sources promoted the exploration of school-level factors, with the former facilitating the exploration of protective factors across time and the latter contributing to the investigation of teaching-related factors.

## 1 Introduction

Academic resilience is commonly attributed to students who demonstrate strong academic performance despite facing adversities. It has gained considerable attention in educational research due to its potential to close the achievement gap and foster social mobility. This construct draws from two theoretical frameworks rooted in psychology and sociology, with the former conceptualizing academic resilience as a personal trait (Masten, 2001; Connor and Davidson, 2003) and the latter emphasizing the interplay between the individual and the context (Luthar et al., 2000; Ungar and Liebenberg, 2011).

Academic resilience is often based on two core components—*risk* or adverse circumstances affecting student progress, and *positive adaptation,* which involves overcoming these challenges and thriving academically (Tudor and Spray, 2017). Such risk factors frequently encompass demographic characteristics (e.g., ethnic identity), available resources (e.g., socio-economic status of the family), interpersonal relationships (e.g., those with peers), health-related and academic hurdles (e.g., school-based discrimination). Conversely, positive adaptations are predominantly associated with beneficial academic outcomes and an enhanced state of wellbeing (Agasisti et al., 2018). Additionally, the concept of academic buoyancy complements academic resilience by addressing how students navigate everyday school-related setbacks and challenges, like exam stress (Martin and Marsh, 2008). These challenges are generally less severe than the adversities investigated in research on academic resilience.

In essence, academic resilience spotlights how disadvantaged students can rise above challenges and succeed academically. A central research objective in this field is to investigate the protective factors that promote academic resilience. This research endeavor can lead to the development of evidence-based interventions tailored to the specific challenges faced by students and a more effective allocation of resources. Such research has implications for informing educational policies that aim to promote equity and reduce disparities in academic achievement.

However, identifying the protective factors that promote academic resilience can be complex due to the variations in theoretical frameworks, operationalization, and research approaches employed across studies. For example, studies that viewed academic resilience as a personal trait typically use an “*instrumental*” operationalization, using scales to measure the construct. These studies typically focus on protective factors closely related to students’ characteristics, such as motivation and engagement (Martin and Marsh, 2008). In contrast, other studies considered the construct as a dynamic progress and often employ *risk* and *positive adaptation* to define academic resilience. The “*simultaneous*” operationalization measures both components at the same time, while the “*progressive*” operationalization assesses positive adaptation sometime after the risk. Studies using these approaches often examine protective factors within the environment, such as school climate (Agasisti et al., 2018).

Even when examining the same protective factor, different studies may adopt various conceptual lenses and concentrate on divergent aspects, thereby leading to inconsistent conclusions. Additionally, the use of advanced statistical methods and complex data sources, such as international large-scale assessments (ILSAs), may introduce confounding factors related to methodological considerations, instrument selection, and level of analysis. Consequently, it becomes challenging to determine whether the differences in the results across diverse studies are due to the varying effectiveness of protective factors in different contexts, or to these variations. Despite the substantial progress made in understanding and measuring academic resilience (Rudd et al., 2021; Ye et al., 2021), there is a lack of literature reviews that specifically examine the protective factors for children and adolescents in educational settings. As a result, less is known about the protective factors that have been extensively studied in education and the extent to which their impacts on academic resilience are consistent across studies.

To address these issues, this review aims to provide an overview of the protective factors that lead to academic resilience and their degree of influence. It focuses on childhood and adolescence, as these developmental periods are crucial for cognitive, social, and emotional development. Early intervention during these phases can improve the long-term prospects for vulnerable students. Moreover, addressing disparities in academic achievement during these formative years can help reduce social inequality (Luthar et al., 2000; Masten, 2001).

## 2 Methods

This study employed a systematic review methodology to search for, identify, and select articles, and subsequently to read, extract, and manage the secondary data collected from those studies. This systematic review approach supports an unbiased and impartial synthesis of the data (Gough et al., 2017). The search process started with identifying research articles to include in the study. We conducted searches for papers in three databases—Eric, PsycINFO, and Web of Science—that include the terms “academic resilienc*” OR “educational resilienc*” OR “academic buoyancy” in the title and abstract. This Boolean search strategy expanded the search criteria and retrieved records that contain any of the specified keywords. The scope of the database search was constrained to English literature published from January 2000 to April 2023. Once articles were found from those searches, they were evaluated based on specific inclusion and exclusion criteria to determine which studies would be included in the final analysis (see section 2.1). Information from the selected studies was then extracted and coded. Results related to operationalization approaches, data sources, and research methods were presented as whole numbers and percentages. The method section is structured to detail each of these steps thoroughly to ensure transparency.

### 2.1 Screening process

A comprehensive search across the three databases yielded 976 pertinent records. This study adhered to the guidelines outlined by Preferred Reporting Items for Systematic Reviews and Meta-Analyses (PRISMA) (McGrath et al., 2017), which provides a detailed framework for conducting a rigorous systematic review. A PRISMA checklist that details our adherence to these guidelines is included in Supplementary Table S4. A three-stage screening process was undertaken involving: screening the title and abstract, assessing the data source and type, and examining the research methods to ensure methodological quality and eligibility.

During Stage 1, papers were screened based on two inclusion criteria: they must contain empirical analysis of data from children or adolescents, and they explore factors associated with academic resilience in educational settings. Empirical studies, which often provide objective and measurable evidence, offer findings that can be replicated and validated. This enhances the reliability of this review and makes the results more applicable to real-world scenarios.

In Stage 2, the screening process included papers from peer-reviewed journals and working papers, while excluding book chapters, conference proceedings, and degree theses. Peer-reviewed journal articles were chosen to ensure confidence in the study quality, and working papers were included to capture the latest research findings and emerging trends, providing a broader perspective on ongoing research in the field (Gough et al., 2017).

**Figure 1:**
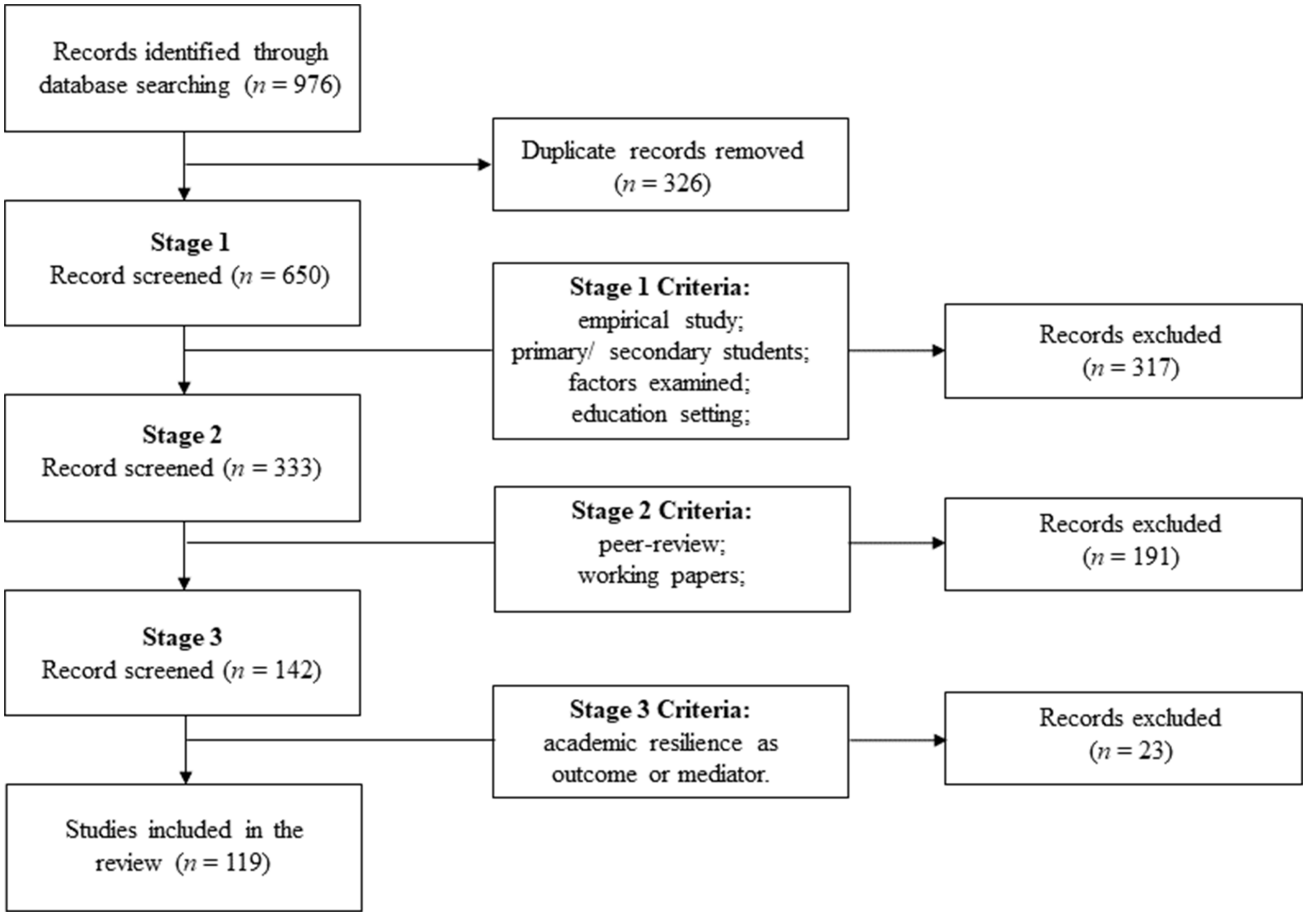
PRISMA diagram of the search process.

For Stage 3, studies were selected for further examination based on a criterion analyzing the impact of various factors on academic resilience. This stage resulted in the inclusion of 119 studies. It is worth noting that articles exploring the reciprocal relationship between academic resilience and protective factors were also included in this review to provide a broader understanding of these factors. The screening was conducted manually, with each of the 119 studies being reviewed by two researchers to ensure they met the inclusion and exclusion criteria listed earlier (Figure 1).

### 2.2 Data analysis

This review examined the interplay between protective factors, operationalizations of academic resilience, data, and research methods. Following the methods of Erlingsson and Brysiewicz (2017), we used a content analysis approach to capture the following information for 119 identified papers:

- the operationalization of academic resilience adopted,
- the data used,
- the factors examined, and
- the research methods employed.

**Figure 2:**
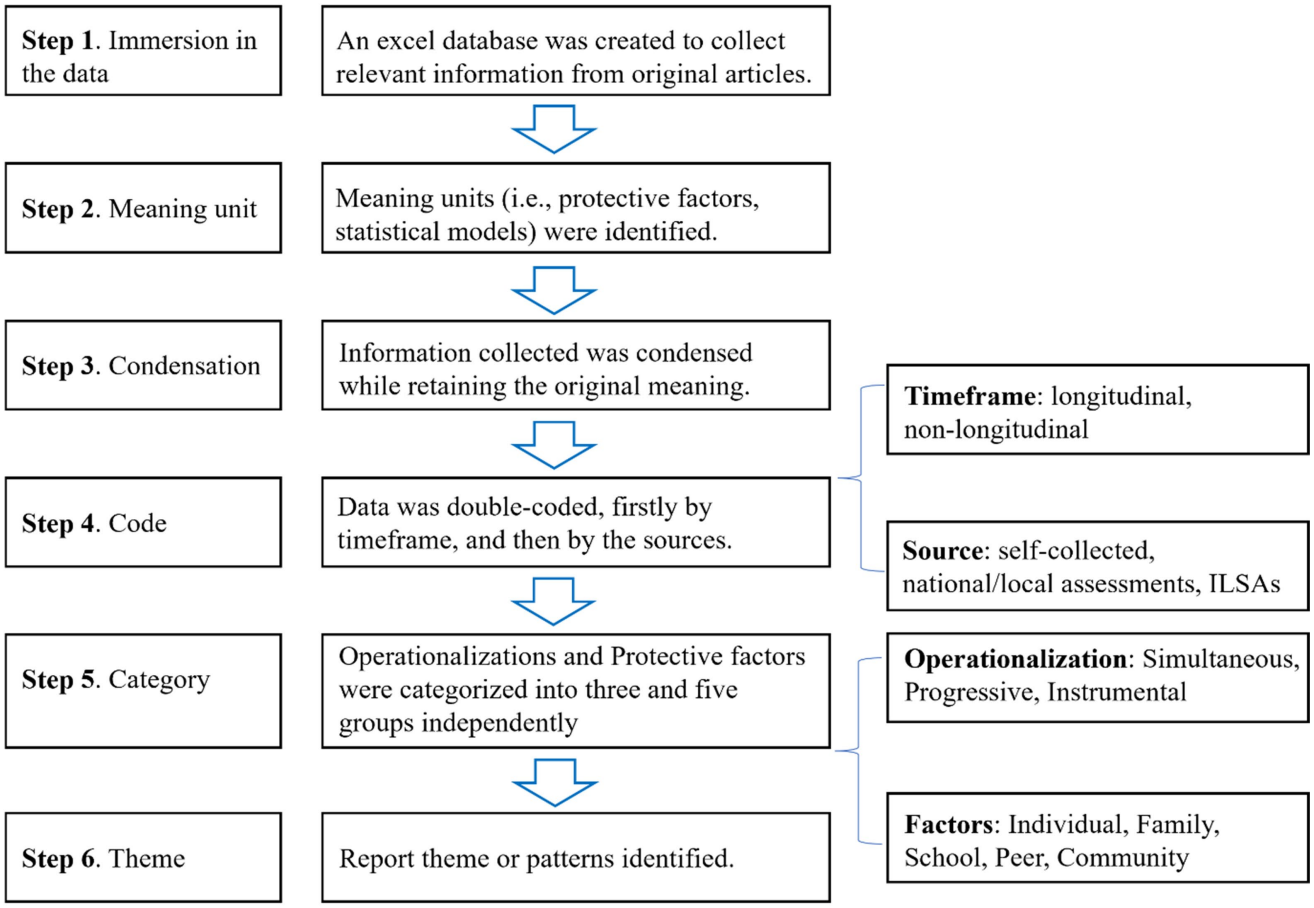
Six-step content analysis.

Content analysis helps address differences between categories and themes, distinguish between levels of abstraction and interpretation, and facilitate the analysis of papers included in this review (Graneheim et al., 2017). The content analysis approach was utilized to examine these four aspects, following a six-step process as outlined in Figure 2. First, an Excel database was created to collect relevant information from original articles. Second, meaning units such as a protective factor or a statistical model were identified. Third, information was condensed while retaining the original meaning. A consistent naming convention was established to ensure that information such as “research method” was accurately and uniformly represented across articles. Fourth, different coding labels were applied to “data” analyzed in these articles, a double-coding procedure was employed. Specifically, the data were coded twice: firstly, by data source (e.g., whether the data were self-collected, derived from national or local assessments, or obtained from international large-scale assessments), and secondly, by time range (e.g., whether the data covered more than one-time point and hence qualified as longitudinal). Fifth, operationalizations and protective factors of academic resilience were grouped into three and five categories, respectively. Finally, we describe the themes or patterns identified through previous coding and categorization efforts. Aggregated information concerning the type of data was expressed in percentages, while those related to operationalization and research methods were presented as whole numbers in the results section.

In examining the operationalization of academic resilience, Rudd et al. (2021) proposed three distinct approaches: (1) *definition-driven* by comparing resilient and non-resilient students, (2) *process-driven* that considers academic resilience as the result of the interactions between risk and protective factors, and (3) *latent construct* by measuring characteristics associated with academic resilience. Nevertheless, Rudd et al. (2021) approaches to operationalize academic resilience did not incorporate temporal influences. Time plays an important role in understanding how protective and risk factors impact the changes in students’ academic resilience over time.

To address this limitation, the present study introduced refined categories for the operationalizations of academic resilience: *simultaneous*, *progressive*, and *instrumental*. The simultaneous approach concurrently assesses the two primary components, namely risk and positive adaptation. In contrast, the progressive approach evaluates risk at an earlier time point and positive adaptation at a later point, offering a dynamic view of how resilience develops and changes. Both approaches acknowledge that the effects of protective factors on outcomes varies over time, while some factors may show immediate effects, others might take longer to manifest. Therefore, the timing of risk measurement may impact researchers’ selection of protective factors and influence their study results. Finally, the instrumental approach uses specific metrics to measure academic resilience, which might manifest as an instrument, a latent variable, or a factor score derived from a scale. By incorporating these categories to operationalize academic resilience, our study aims to better understand its protective factors by considering the dynamics in student adaptations and success.

Diverse theoretical frameworks have influenced the focus on protective factors in resilience research. Werner (2000), for instance, conceptualized resilience from a personal trait perspective and categorized protective factors into distinct developmental periods: infancy (e.g., sociability), childhood (e.g., positive self-concept), and adolescence (e.g., planning). Conversely, Cutuli et al. (2016) emphasized the dynamic interaction between individual and their contexts, organizing protective factors into multiple categories: individual characteristics (e.g., self-regulation), family and close relationships (e.g., good parenting), community and connections with organizations (e.g., effective schools). Given the educational context of this study, which places a stronger emphasis on malleable factors like school resources to promote resilience, this study adopted Cutuli et al’s categorization (Cutuli et al., 2016). Protective factors are divided into five groups: individual, family, peer, school, and community. It is worth noting that these factors present at multiple levels when one considers the hierarchical structure (e.g., students within schools). However, analytical approaches vary across studies, with some potentially overlooking these nested structures. Therefore, this study will address these five groups of protective factors independently, delving deeper into their analyses in the research methods section.

## 3 Results

**Figure 3:**
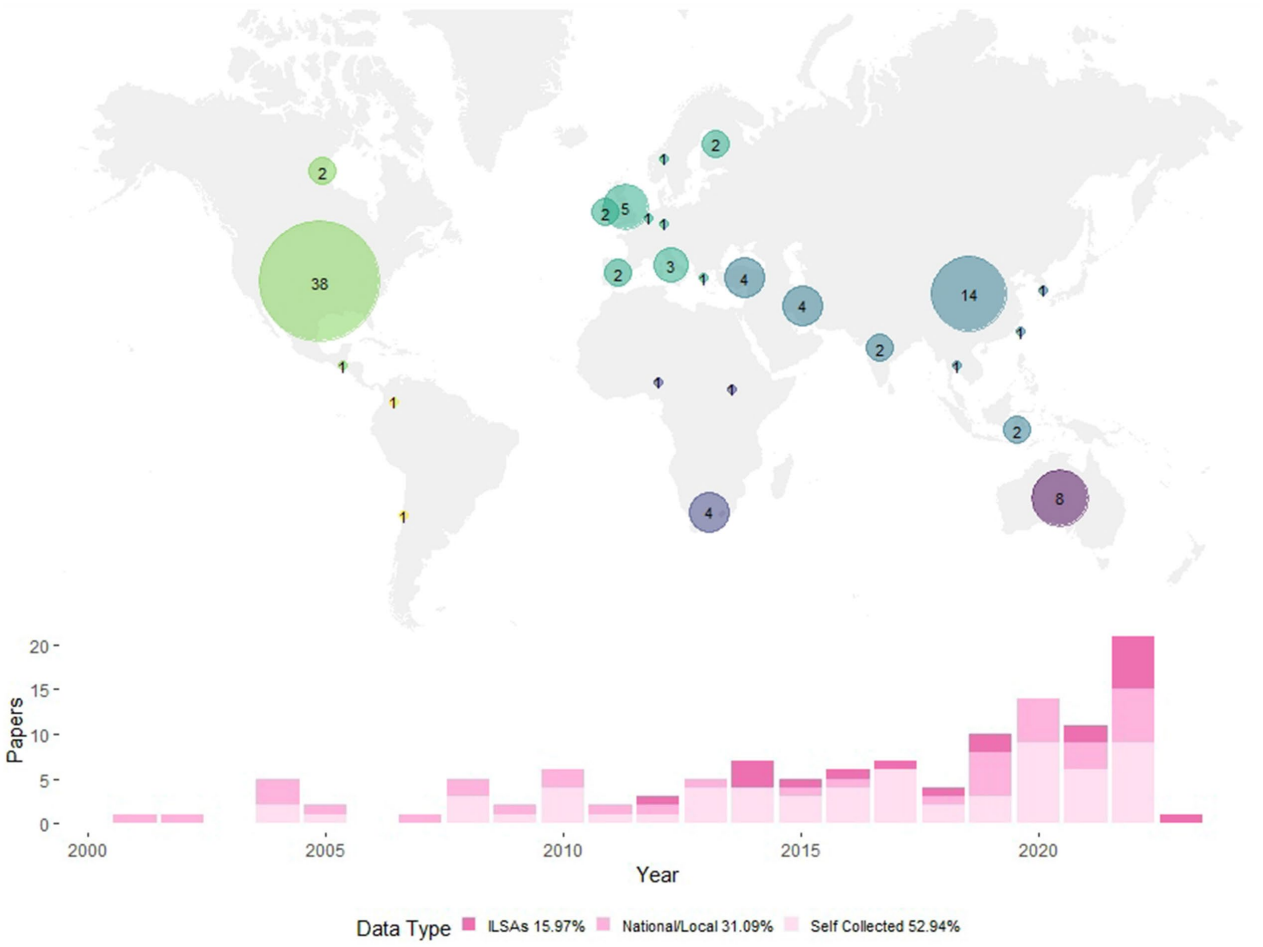
Overview of papers included. Fifteen papers examining multiple countries were not displayed on the map.

Among the 119 studies analyzed in this review, the majority were carried out in the United States (31.93%), China (11.76%), and Australia (6.72%). Specifically, out of the 32 studies conducted before 2013, 22 were from the United States. However, since 2013, more countries have engaged in research on academic resilience, indicating a growing global interest and awareness of this construct. Here, we systematically review protective factors associated with academic resilience, including research methods employed, the data analyzed, and the definitions utilized for academic resilience. It should be noted that this research does not constitute a meta-analysis. As such, it does not offer a single regression coefficient that would aggregate the findings from all studies for each protective factor or provide a comprehensive range of these coefficients. Additionally, due to considerable analytical variation among the studies, comparing regression coefficients across them presents a significant challenge, especially when the operationalizations of academic resilience varied significantly (Figure 3).

### 3.1 Protective factors associated with academic resilience

**Figure 4:**
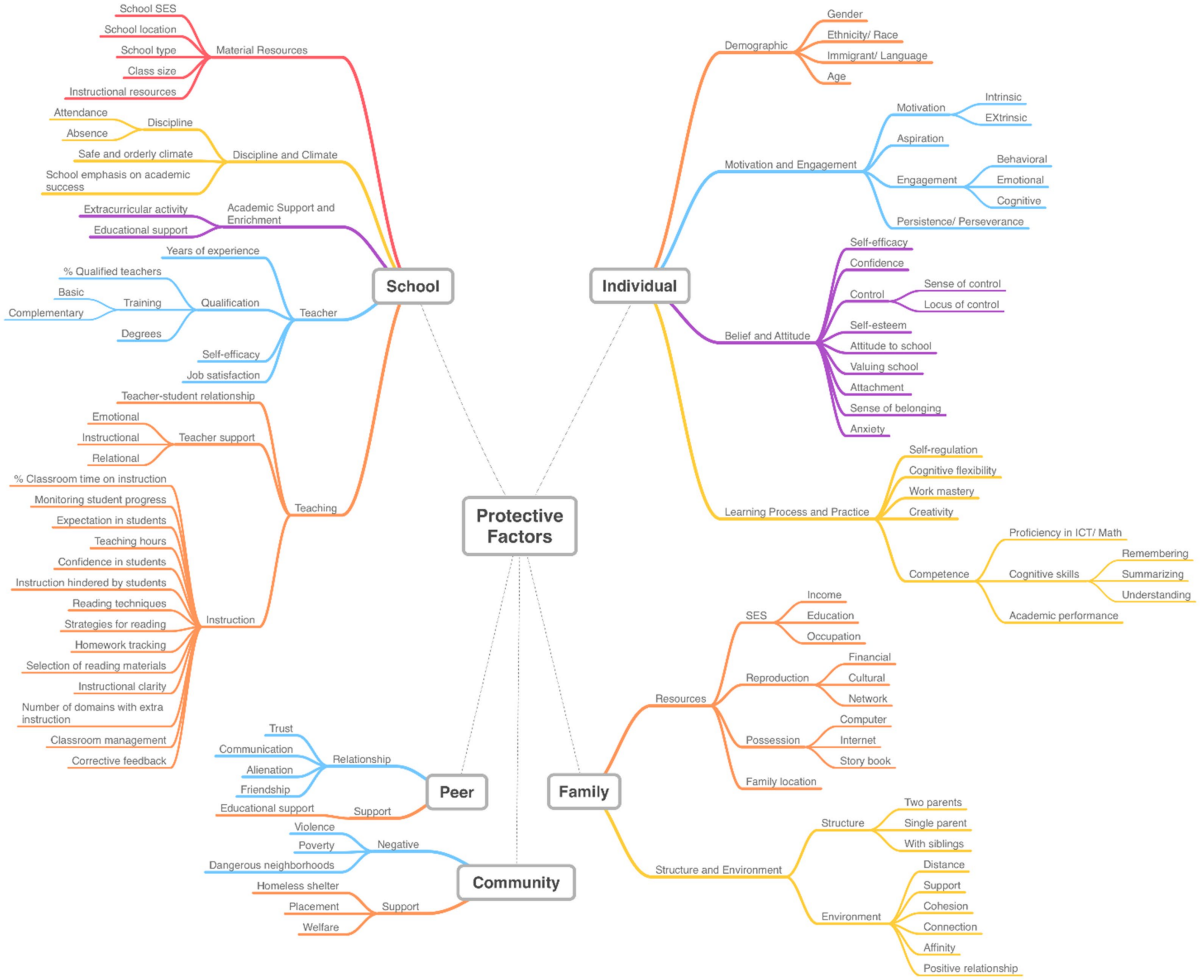
Protective factors discussed in the literature.

The 119 analyzed articles yielded 764 factors. Individual factors constitute 50% of the total, while school factors comprise 30.24%. Family factors account for 14.66%, whereas peer and community factors represent 2.88 and 2.22%, respectively. We organized the protective factors into sub-categories for each group and subsequently presented the associated themes (Figure 4).

#### 3.1.1 Individual

##### 3.1.1.1 Demographic factors

Demographic variables, such as race/ethnicity, immigration status, and age, typically show a negative correlation with academic resilience. Minority status, immigration, and older age are often relate to lower levels of resilience. However, gender does not follow this pattern.

Gender has been the subject of extensive empirical investigation, with inconsistent findings reported across studies. Yavuz and Kutlu (2016) observed that female students demonstrated greater levels of academic resilience when compared to male students. Similarly, Kong (2020) found that a positive parent–child relationship was associated with greater resilience among low-SES female students. However, several studies have failed to detect gender differences in academic resilience (Miller et al., 2013; Tope-Banjoko et al., 2020).

Schelble et al. (2010) and Çelik (2017) found a significant association between ethnicity and academic resilience. This association has been extensively studied in racially diverse countries such as the United States (Langenkamp, 2010). In general, immigrant students have been observed to exhibit lower levels of resilience (De Feyter et al., 2020). Nevertheless, a contradictory finding was reported by Cheung et al. (2014) in Singapore.

Age serves as a control variable in longitudinal studies to explore relationships over time (Martin et al., 2010) and is also used as a predictor of academic resilience. Older students are found to have lower academic resilience, potentially due to an increased likelihood of grade repetition (Wills and Hofmeyr, 2019). Hofmeyr (2019) confirmed that high-achieving students in Progress in International Reading Literacy Study (PIRLS) and Trends in International Mathematics and Science Study (TIMSS) tend to be younger, with a greater age disparity in TIMSS than in PIRLS. However, Armfield et al. (2021) reported a contrasting finding, whereby being older was a predictor of academic resilience among maltreated children.

##### 3.1.1.2 Motivation and engagement factors

Significant associations between academic resilience and factors related to motivation and engagement were not universally identified across countries. This inconsistency may be partially explained by the presence of limited statistical power resulting from small cluster sizes, particularly in ILSAs data.

Most research on motivation has focused on intrinsic and extrinsic factors, with only a handful of studies exploring general motivation (Yu and Martin, 2014; Bostwick et al., 2022). Scholars have dedicated considerable attention to investigating intrinsic motivation factors, including students’ enjoyment of learning (Cheung et al., 2014). Likewise, extrinsic motivation factors, such as motivation for academic success, have received extensive research attention (Cui et al., 2022), making them the most commonly studied motivational factors.

Agasisti and Longobardi (2012) and Süleyman (2022) identified both intrinsic and extrinsic motivation factors as facilitators of academic resilience. In addition, Baniani and Davoodi (2021) found that achievement motivation can explain up to 15% of students’ academic resilience. However, contrasting findings were also identified. Vicente et al. (2021) compared three aspects of motivation between high-income and low-income countries, including motivation to work, reading enjoyment, and career expectations. They found that reading enjoyment and career expectations promote academic resilience in both groups, while motivation to work only predicts academic resilience in high-income countries. Moreover, Garcia-Crespo et al. (2022) examined the influences of students’ enjoyment in learning mathematics and science on academic resilience but only found a significant association in mathematics.

Student aspiration, a construct closely intertwined with motivation, has been identified as a salient factor that promotes academic resilience, as evidenced in prior research (Sacker and Schoon, 2007; Gizir and Aydin, 2009). Erberer et al. (2015) reported that students’ educational aspirations were the most potent and reliable predictor of academic resilience, with significant correlations observed in 20 out of 28 economies examined. Additionally, Kong (2020) found that aspiration significantly predicts higher academic achievement, with a stronger association observed among disadvantaged students.

Motivation and aspiration provide the initial impetus for goal pursuit, while sustained engagement in activities that align with one’s goals is crucial in promoting academic resilience. Specifically, engagement is a significant predictor of academic resilience and can mediate the association between risk factors and student outcomes (Jang et al., 2023). Despite the importance of engagement in academic resilience, the relationship between the two factors may differ when examined across different countries. Garcia-Crespo et al. (2019) found a significant association between engagement and academic resilience in 11 out of 23 countries studied. In addition, Kothari et al. (2021) investigated the association between academic resilience and three aspects of engagement: behavioral, emotional, and cognitive. And they found that behavioral engagement predicts students’ resilience in mathematics and reading, while emotional engagement is positively related to students’ attendance.

Two other concepts closely associated with engagement are persistence and perseverance, both of which have been shown to correlate positively with academic resilience (Collie et al., 2017a,b; Wills and Hofmeyr, 2019). However, these concepts have received less attention in the field than engagement.

##### 3.1.1.3 Belief and attitude factors

With the exception of self-esteem and attitude toward school, protective factors related to students’ beliefs and attitudes, including self-efficacy, confidence, sense of control, and valuing of school, consistently exhibit a positive association with academic resilience.

Self-efficacy is a well-studied protective factor in academic resilience, with a consistently positive relationship documented in numerous studies (Borman and Overman, 2004; Victor-Aigboidion et al., 2020). Moreover, research has explored two specific types of self-efficacy, namely academic and computer self-efficacy, and found that they were instrumental in promoting academic resilience. Additionally, some scholars have reported that self-efficacy indirectly affects test anxiety through academic buoyancy (Lei et al., 2022).

Confidence is a frequently studied protective factor that is closely related to self-efficacy. Hofmeyr (2019) found that self-confidence was the most powerful predictor of academic resilience across different domains (reading, mathematics, and science) and grade levels (fourth and ninth grades). Similarly, Garcia-Crespo et al. (2021) found that confidence was significantly and positively associated with academic resilience in all 23 countries examined. In addition, significant associations were found between academic resilience and confidence in both mathematics and science (Garcia-Crespo et al., 2022).

Locus of control, a protective factor originating from early resilience studies in psychology (Werner, 2000), was found to predict academic resilience (Borman and Overman, 2004; Morales, 2008). In addition, Cappella and Weinstein (2001) found that the high school curriculum partly mediated the relationship between internal locus of control and academic resilience. Some studies examined the relationship between academic resilience and the sense of control across time. Martin (2013) reported that academic buoyancy measured at an earlier time point was a significant negative predictor of uncertain control at a later time point. Collie et al. (2015) found bidirectional relationships between academic buoyancy, achievement, and sense of control across time.

Self-esteem has received less research attention compared to the aforementioned factors. While Cappella and Weinstein (2001) found no significant association between self-esteem and academic resilience, other investigations have reported a positive and significant relationship (Wayman, 2002; Cunningham and Swanson, 2010).

A group of protective factors linked to students’ attitudes, attachment, and values toward education and the school have been identified. Empirical studies have reported mixed results on the associations between academic resilience and students’ attitudes toward school. Some studies, such as Wayman (2002) and Borman and Overman (2004), have found a positive association, whereas others, such as those conducted by Agasisti and Longobardi (2012) in Italy and Wills and Hofmeyr (2019) in South Africa, have reported no significant predictive power. On the contrary, students’ valuing of school was significantly related to academic resilience (Gayles, 2005; Collie et al., 2017a,b). Moreover, research has also identified students’ attitudes toward computers and mathematics as significant predictors of academic resilience (Agasisti and Longobardi, 2012).

Recent academic resilience studies have demonstrated growing attention toward investigating the role of students’ attachment and sense of belonging to their schools. Although no significant association was found between school attachment and academic resilience (Yavuz and Kutlu, 2016), research indicates that students’ sense of belonging to the school significantly predicts academic resilience (Rosen et al., 2019).

Several factors negatively associated with academic resilience have been identified in the literature, with students’ anxiety being particularly well-studied. Empirical investigations have consistently reported a significant negative correlation between anxiety and academic resilience (Fiorilli et al., 2020; Mohan and Kaur, 2021). Additionally, some scholars have found that test anxiety is a significant negative predictor of academic resilience (Lei et al., 2021).

##### 3.1.1.4 Learning progress and practice factors

Protective factors associated with students’ learning progress or practice have received relatively limited research attention. Among these factors, self-regulation has consistently emerged as a predictor of academic resilience.

Empirical research has established a positive association between students’ self-regulation and academic resilience, as evidenced by several studies (Kumi-Yeboah, 2020; Koirikivi et al., 2021). In further examining the construct of self-regulation, Nota et al. (2004) found positive relationships between both cognitive and motivational self-regulation and academic resilience.

The relationship between cognitive flexibility and academic resilience remains inconclusive in the literature. Yavuz and Kutlu (2016) reported a positive association, while Süleyman (2022) found the opposite pattern in Turkey. Mixed findings regarding the relationship between work mastery and academic resilience have been reported. While Özcan and Bulus (2022) reported a significant positive association, Süleyman (2022) found no significant relationship between the two constructs. In contrast, creativity was positively related to academic resilience (Chen et al., 2018; Chen and Padilla, 2022).

The literature has also explored protective factors related to students’ competence, including their proficiency in information and communication technology and reading, which have been found to predict academic resilience (Özcan and Bulus, 2022). Additionally, students’ cognitive skills, such as remembering, summarizing, and understanding, have been positively associated with academic resilience (Süleyman, 2022). Longitudinal studies have also shown that students’ academic performance at an early stage is positively related to academic resilience (Rosen et al., 2019; Zaw et al., 2022).

#### 3.1.2 School

##### 3.1.2.1 School material resources

The relationship between school material resources and academic resilience is contingent upon the specific measurement instrument employed. For example, measures such as school average socioeconomic status (SES) and location consistently exhibit predictive capabilities for academic resilience. In contrast, factors such as school type, class size, and instructional resources yield varying and inconclusive outcomes.

School socioeconomic status (SES) is frequently investigated as one of the prominent school resource factors in relation to academic resilience, often operationalized by the average SES of the enrolled students. Most studies investigating this variable have utilized data from ILSAs (Agasisti et al., 2018) or have adopted the SES calculation methods used by these assessments (Cui et al., 2022), which typically rely on items such as parents’ education, occupation, and home possessions. Consistent results have been identified in studies examining the relationship between school SES and academic resilience, demonstrating a significant positive association between the two factors (Wills and Hofmeyr, 2019; Vicente et al., 2021). Hofmeyr (2019) also investigated the association between school SES and academic resilience in TIMSS and PIRLS, with the finding showed more pronounced relationship in TIMSS study.

Empirical research indicates that school location, irrespective of whether measured by suburban or urban location or population density of the area where the school is situated, emerges as a significant predictor of academic resilience (Agasisti and Longobardi, 2012; Ge and Ngai, 2020). Conversely, school type, such as public or private, does not constitute a reliable predictor of academic resilience (Agasisti and Longobardi, 2012; Vicente et al., 2021).

Class size is commonly discussed in the literature, but most studies examined have not found a significant relationship between class size and academic resilience (Borman and Overman, 2004; Wills and Hofmeyr, 2019; Vicente et al., 2021). However, Agasisti et al. (2018) have reported a positive relationship between larger class sizes and academic resilience, which may be explained by compensation policies that provide larger classes with more experienced teachers. Similar to class size, the influence of the computer-student ratio on academic resilience appears to be non-significant (Agasisti et al., 2018). Academic resilience was also investigated with school instructional resources; however, the relationship between the two is inconclusive (Borman and Overman, 2004; Agasisti et al., 2018).

##### 3.1.2.2 School climate and discipline

Discipline and climate-related protective factors, including disciplinary practices, a safe and orderly environment, and a school emphasis on academic success, consistently predict academic resilience. Discipline-related factors, notably student attendance or absence, are significantly related to academic resilience (Thiessen, 2008; Fantuzzo et al., 2012; Hofmeyr, 2019). Nonetheless, certain studies have reported insignificant associations. Gabrielli et al. (2022) identified significant associations between discipline climate and academic resilience among immigrant students in most European countries, except for the United Kingdom. However, Garcia-Crespo et al. (2022) found no significant association between discipline climate and academic resilience in European Union member countries.

Empirical studies have proved a positive relationship between a safe and orderly climate and academic resilience (Borman and Overman, 2004; Garcia-Crespo et al., 2021; Koirikivi et al., 2021). Nevertheless, contradictory findings are observed when the correlation is scrutinized across various nations. Sandoval-Hernández and Bialowolski (2016) found significant associations between a safe and orderly climate and academic resilience in only half of the East Asia economies examined.

Likewise, a positive relationship exists between school emphasis on academic success and academic resilience, although the strength of the association varies across countries (Erberer et al., 2015; Sandoval-Hernández and Bialowolski, 2016), which may be partially attributed to insufficient statistical power resulting from small cluster sizes. Notably, most studies that investigated the safe and orderly climate and emphasis of schools on academics have relied on data from TIMSS or PIRLS.

##### 3.1.2.3 School academic support and enrichment

Protective factors associated with school academic support and enrichment exhibit a generally positive association with academic resilience, encompassing activities beyond the curriculum and supplementary academic assistance. The positive association between extra-curricular activities and academic resilience has been consistently demonstrated in research (Randolph et al., 2004; Agasisti and Longobardi, 2012). Notably, some scholars consider a school’s capacity to provide extra-curricular activities as an indicator of its available resources (Agasisti and Longobardi, 2014). However, some scholars have adopted a comprehensive viewpoint by investigating students’ extra-curricular activities within and beyond the school setting. For example, Peck et al. (2008) investigated the relationship between academic resilience and extra-curricular activities, such as sports, reading, chores, and music.

Additionally, educational support provided by schools is also studied with academic resilience. Cappella and Weinstein (2001) found that structural support during the transition to high school positively predicts academic resilience. Bester and Kuyper (2020) discovered that additional academic support in schools, including more tuition time, enhances academic resilience. In addition to fostering academic resilience, educational support has been found to positively impact students’ wellbeing (Oldfield et al., 2020). Longitudinal studies also found small and positive associations between academic resilience and school learning support across time (Bostwick et al., 2022).

##### 3.1.2.4 Teacher factors

Teacher quality, often assessed as an indicator of school human resources, is a frequently investigated variable. However, the influence of this factor on academic resilience appears to be contingent upon the particular metric employed for its assessment. Teacher quality, as indicated by factors such as years of experience, training, and degree attainment, yields mixed findings regarding its impact on academic resilience. However, student-teacher ratio and teacher shortage have been identified as predictors of academic resilience.

Borman and Overman (2004) found that teachers’ years of experience do not predict academic resilience. However, Agasisti et al. (2018) reported a significant association between teachers’ years of experience at a specific school and academic resilience but not with their overall years of experience.

Garcia-Crespo et al. (2021) investigated teachers’ basic training and complementary training in 23 countries, with the former predicting academic resilience in two and the latter showing a positive relationship in eight countries. Similarly, Wills and Hofmeyr (2019) reported that the proportion of language teachers with language specializations predicts academic resilience. Additionally, Agasisti and Longobardi (2012) found that a school’s ratio of qualified teachers predicts academic resilience. On the contrary, Vicente et al. (2021) found that properly trained teachers in a school do not significantly influence academic resilience.

Jin et al. (2022) employed the proportion of teachers possessing a bachelor’s or master’s degree as a metric for teacher quality, finding a positive correlation with academic resilience in China. However, Hofmeyr (2019) did not find a significant relationship between teachers’ degrees and academic resilience in South Africa. Furthermore, Agasisti and Longobardi (2012) investigated the influence of teacher-student ratios and teacher shortage, finding both factors significantly predict academic resilience.

Teacher factors, such as self-efficacy and job satisfaction, have been investigated in relation to academic resilience, with self-efficacy showing a positive association and job satisfaction having mixed findings. For instance, Garcia-Crespo et al. (2021) utilized PIRLS data and found that job satisfaction predicted academic resilience in four out of 23 countries. Whereas in a follow-up study, Garcia-Crespo et al. (2022) employed TIMSS data and did not find such a relationship in either mathematics or science.

##### 3.1.2.5 Teaching factors

Protective factors linked to teaching primarily revolve around the quality of teacher-student relationships and instructional effectiveness. Teacher-student relationships have received extensive research attention, while instructional effectiveness has gained prominence, particularly with the advent of ILSAs. The former has shown a positive correlation with academic resilience, while the latter has yielded inclusive findings.

Except for one study, all examined papers investigating the influence of teacher-student relationships found a positive prediction of academic resilience (e.g., Langenkamp, 2010; Cui et al., 2022). Crosnoe and Elder (2004) examined non-parental relationships and reported that teacher bonding has the strongest impact on students’ off-track behavior. Similarly, Strolin-Goltzman et al. (2016) found that teacher-student relationships were the most powerful influence on students’ educational success. Özcan and Bulus (2022) adopted two items to measure teacher-student relationships: teachers’ stimulation of reading engagement and teachers’ interest perceived by students. However, their study did not find a significant association between these measures and academic resilience.

Teacher support, closely related to teacher-student relationships, is also examined with academic resilience in the literature. However, the finding is mixed. Neal (2017) and Fang et al. (2020) reported a significant relationship between teacher support and academic resilience. However, Bostwick et al. (2022) found little to no association between teachers’ relational support and academic buoyancy. Furthermore, Granziera et al. (2022) examined the impact of teachers’ emotional and instructional support, discovering that while the former predicts academic resilience, the latter does not.

Protective factors associated with teaching quality received comparatively less research attention before the emergence of ILSAs. Borman and Overman (2004) examined the relationship between academic resilience and two variables related to teaching: the percentage of classroom time dedicated to instruction and the monitoring of student progress. Although they did not discover a significant relationship between either of these variables and academic resilience, they identified that the former variable positively impacted academic resilience in minority students. Additionally, Schoon et al. (2004) found that teachers’ expectations were the most robust predictor of academic resilience.

Using PISA data, Agasisti and Longobardi (2012) established a positive correlation between the number of teaching hours per year and academic resilience. Similarly, Erberer et al. (2015) employed TIMSS data and determined that teachers’ confidence in their students is the most consistent predictor of academic resilience at the school level. Garcia-Crespo et al. (2021) analyzed PIRLS data from 23 countries and found that classroom instruction hindered by disruptive behavior was predictive of academic resilience in eight countries, while comprehensive or reflective reading techniques were predictive in seven countries. Routine or systematic strategies for reading, homework tracking, and selection of adapted reading showed a lower predictive capacity for academic resilience. Furthermore, Garcia-Crespo et al. (2022) employed TIMSS data and found that students’ perception of instructional clarity predicts their academic resilience in both mathematics and science. Jin et al. (2022) used PISA data and determined that the number of learning domains with additional instruction negatively predicts academic resilience.

Research using data other than ILSAs also examined teaching quality factors with academic resilience. Bostwick et al. (2022) found that classroom management predicts academic buoyancy 1 year later. Reported that teachers’ corrective feedback promotes academic resilience.

#### 3.1.3 Family

##### 3.1.3.1 Family resources

Family resources, particularly family SES, are commonly employed to characterize risk status, such as low SES. However, in some studies, family SES, typically derived from parents’ income, education, and occupation, is also investigated as a protective factor in academic resilience for students with risks of dropping out, demonstrating low reading proficiency, and belonging to minority groups. The majority of research examined revealed a positive and statistically significant correlation between family SES and academic resilience (Cappella and Weinstein, 2001; Li and Yeung, 2019). Several studies have investigated the association between academic resilience and parental education or work, albeit with inconsistent findings (Borrett and Rowley, 2020).

Bussemakers and Kraaykamp (2020) adopted a reproduction perspective and reported a significant association between academic resilience and family cultural and financial resources. Similarly, Çelik (2017) established a positive relationship between academic resilience and parents’ network. The relationship between home possessions, including computers, internet connections, and story books, and academic resilience has been investigated in the literature. However, the findings on these factors are inconclusive and inconsistent (Thiessen, 2008; Wills and Hofmeyr, 2019). Family location predicts academic resilience, especially in rural areas (Kong, 2020; Armfield et al., 2021).

##### 3.1.3.2 Family structure and environment

Protective factors related to family structure, such as a single parent or with siblings, are generally not significantly associated with academic resilience (Cunningham and Swanson, 2010; Cheung et al., 2014). In contrast, a family with two parents positively influences academic resilience (Thiessen, 2008; Langenkamp, 2010).

Family environment, especially a positive parent–child relationship, has been found to predict academic resilience in most papers examined (Pan and Yi, 2011; Paat, 2015; Oldfield et al., 2020). However, Chen et al. (2018) and Li and Yeung (2019) found no such relationship in China.

##### 3.1.3.3 Academic support and involvement

Research has indicated a positive association between academic resilience and parental educational support or involvement, e.g., communication with teachers (Schoon et al., 2004; Anagnostaki et al., 2016; Bester and Kuyper, 2020). Reading to children has been found to predict academic resilience (Armfield et al., 2021). However, checking homework is not significantly associated with academic resilience (Li and Yeung, 2019), and helping with homework is negatively related to academic resilience (Wills and Hofmeyr, 2019). Additionally, educational investment is positively related to academic resilience (Li and Yeung, 2019; Austin et al., 2022).

Several scholars have identified parents’ educational expectations as a significant predictor of academic resilience (Gizir and Aydin, 2009; Pan and Yi, 2011). However, contradictory evidence has been presented by other scholars. Crosnoe and Elder (2004) and Li and Yeung (2019) reported that parents’ expectations do not predict academic resilience.

#### 3.1.4 Peer and community

##### 3.1.4.1 Peer relationships and supports

Academic resilience and its association with peers have primarily been discussed in two distinct categories: peer relationships and peer support. While the impact of peer relationships on academic resilience is inconclusive, studies suggest that friendship is a significant predictor of academic resilience (Bellis et al., 2018). On the other hand, peer support, which encompasses educational assistance, is positively associated with academic resilience (Thiessen, 2008; Koirikivi et al., 2021). For example, Chen et al. (2018) have operationalized the construct of peer support through three dimensions: peer trust, peer communication, and peer alienation, and reported a significant association between peer support and academic resilience.

##### 3.1.4.2 Community factors

Community factors have received relatively limited attention in the literature. Early studies have established a link between the community and ethnic identity, which may be reinforced through community interaction, and subsequently function as a protective factor for academic resilience (Gayles, 2005). Several scholars have explored community-level factors negatively correlated with academic resilience, such as community violence, poverty, and dangerous neighborhoods (Çelik, 2017; Ge and Ngai, 2020). Rachmawati et al. (2021) reported a positive relationship between community support and academic resilience, while Yavuz and Kutlu (2016) found no significant association between academic resilience and students’ perception of social support. Community support factors, such as homeless shelters and the number of placements, have not been found to predict academic resilience (Fantuzzo et al., 2012; Strolin-Goltzman et al., 2016). However, receiving public assistance, such as welfare, has been identified as a predictor of academic resilience (Nichols et al., 2016).

### 3.2 Operationalization and data used in the literature

This review identified three approaches for operationalizing academic resilience: (1) *simultaneous*, which measures both risk and positive adaptation at the same time; (2) *progressive*, which measures risk at an earlier time point and positive adaptation at a later time point, treating academic resilience as a developing process over time; and (3) *instrumental*, which measures academic resilience using multiple items or scales. Table 1 displays a comparable utilization of the “simultaneous” and “instrumental” approaches, amounting to approximately 36%. Conversely, fewer articles utilized the “progressive” approach, accounting for roughly 25.21%. Three articles have failed to clearly define academic resilience, resulting in the inability to classify their operationalizations and label them as “Not Available (NA).” Supplementary Tables S1–S3 provide a comprehensive summary of the studies utilizing these operationalizations of academic resilience.

**Table 1:** Data and operationalizations of academic resilience.

		Data source		
Operationalization	Time range	ILSAs 19	National/Local 37	Self-collected 63
Simultaneous (44)	Longitudinal	0	1	0
	Non-longitudinal	19	7	17
Progressive (29)	Longitudinal	0	21	4
	Non-longitudinal	0	1	3
Instrumental (43)	Longitudinal	0	2	8
	Non-longitudinal	0	4	29
NA (3)	Longitudinal	0	0	0
	Non-longitudinal	0	1	2

NA, operationalization not available.

Considering the data source, a significant proportion of the studies (52.94%) collected their own data. In contrast, approximately 31.09% relied on pre-existing national or local assessments, and 15.97% utilized data from ILSAs. Furthermore, ILSAs data has become increasingly prevalent in the literature since the 2010s, particularly in comparative analyses across different countries. Of the 15 studies that investigated academic resilience across countries, 14 used data from ILSAs. Of the 119 reviewed articles, 36 included longitudinal data collected at multiple time points.

Concerning their operationalizations for academic resilience, studies utilizing “simultaneous” and “progressive” approaches exhibited divergent tendencies. All studies employing a “simultaneous” method were conducted using non-longitudinal data except for one. This particular article utilized longitudinal data from kindergarten to 4th grade. Notwithstanding, the analysis was conducted for each individual grade, where both risk and positive adaptation were assessed at the same time (De Feyter et al., 2020). In contrast, all studies adopting a “progressive” approach used longitudinal data except for four. Despite the absence of longitudinal data in these articles, they employed retrospective or follow-up designs that require participants (e.g., former foster youth) to reflect on their experiences and establish a connection between their past and current circumstances (Neal, 2017; Bussemakers and Kraaykamp, 2020). In research that employed an “instrumental” approach, longitudinal data were often utilized to scrutinize the reciprocal connections between academic resilience and its associated factors (Martin et al., 2010).

### 3.3 Research methods employed in the literature

#### 3.3.1 Mixed or qualitative methods

Four mixed-methods studies employed qualitative research techniques, such as interviews and classroom observations, followed by the use of quantitative methods, such as analysis of variance (ANOVA), multivariate analysis of variance (MANOVA), correlation, and regression analysis (Nota et al., 2004; Rivera et al., 2012; Strolin-Goltzman et al., 2016; Austin et al., 2022).

**Figure 5:**
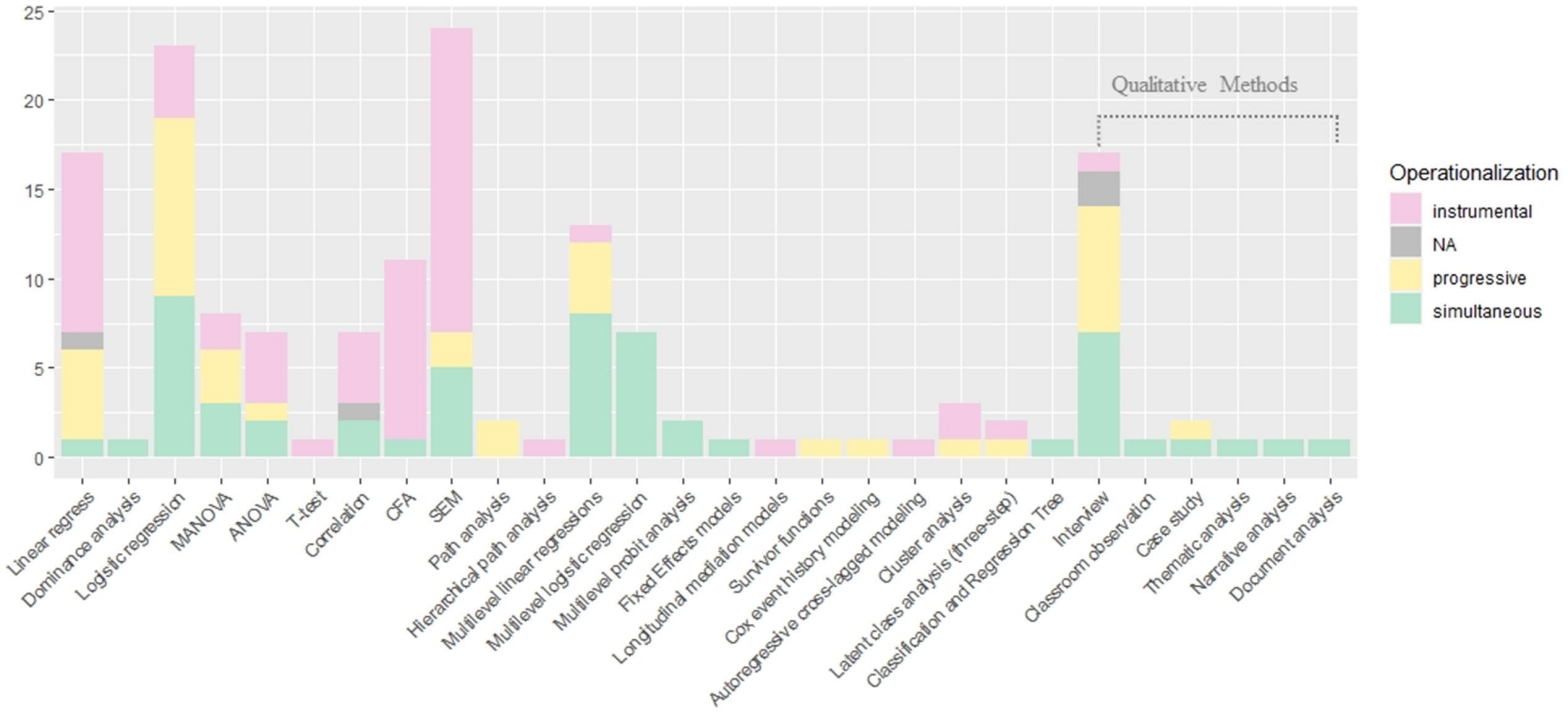
Research methods and operationalizations. Some studies reviewed in this analysis utilized multiple main methods, resulting in more methods being used than the total number of papers reviewed.

In the 15 qualitative studies reviewed, data was often collected using open-ended and semi-structured interview techniques (Gayles, 2005; Çelik, 2017). In cases where researchers sought to concentrate on a limited number of students and obtain in-depth information, case study methods were utilized (Rojas Flórez, 2015; Watson and Vogel, 2017). Researchers employed ethnographic interviews and image elicitation methods to address cultural differences, such as those related to African American students and Inuit youth (Morales, 2008). Furthermore, follow-up methods were used to assess the influence of protective factors over time (Morales, 2008; Kumi-Yeboah, 2020). Researchers also interviewed students, parents, and teachers in several studies (Pan and Yi, 2011; Graves, 2014). For instance, Çelik (2017) interviewed both resilient and non-resilient students along with their mothers. In analyzing the data collected from these interviews, researchers employed narrative, document, and thematic analyses (Gayles, 2005; Rojas Flórez, 2015; Figure 5).

#### 3.3.2 Quantitative methods

##### 3.3.2.1 Simultaneous

Of the 44 studies using simultaneous operationalization, 35 utilized quantitative methods (see Supplementary Table S1). Most of these studies treated academic resilience as a binary outcome (e.g., resilient or not), while about 30% of them operationalized it as a continuous variable (e.g., percentage of resilient students in a school). Subsequently, both logistic and linear regressions were frequently used in the literature.

Except for several studies (Erberer et al., 2015; Sandoval-Hernández and Bialowolski, 2016; Hofmeyr, 2019), most research considered the nesting structure, recognizing that students from the same school may share more similarities. Scholars used multilevel models such as hierarchical linear/logistic regression, two-level structural equation models with random intercepts, or one-level regressions considering data clustering (Cunningham and Swanson, 2010; De Feyter et al., 2020; Ge and Ngai, 2020).

Despite recognizing the nesting structure, some scholars have employed one-level regression to analyze variables from the same level without accounting for the multilevel structure to obtain a sparse model. For instance, Cheung et al. (2014) and Cheung (2017) focused solely on protective variables at the student level, while Li and Yeung (2019) investigated the influence of individual, family, and school protective factors on academic resilience in three separate regressions. However, these methods failed to account for the non-independence of students within the same school or class, which may result in biased estimates.

Academic resilience studies using multilevel modeling commonly employ a stepwise approach, wherein a series of models are fitted, starting with a baseline model that includes student-level variables only, and gradually adding classroom or school-level factors until a final model is obtained that contains variables from all levels (Agasisti et al., 2018). This process allows for the examination of the contribution of each level of analysis to the outcome and facilitates the identification of the most critical factors associated with academic resilience. However, the covariances between protective factors and their interactions across different levels are scarcely addressed in the literature, particularly in studies that utilize ILSAs data and examine numerous protective factors across nations.

Various approaches have been utilized to address the challenge of examining interactions among multiple protective factors. For example, Kosciw et al. (2015) employed multi-group structural equation models to investigate the influence of protective factors on academic resilience in urban, suburban, and rural areas. In multilevel modeling studies, demographic variables are typically incorporated as control variables, and interactions between covariates are usually assessed at a designated stage of the analysis. For example, Anagnostaki et al. (2016) investigated protective factors in six successive steps and examined interactions at each step. Nevertheless, this method has limitations in cross-national comparisons, as using the same model across different countries may not account for variations in interactions among the variables.

Studies have demonstrated a preference for certain protective factors based on the assessment design of ILSAs data utilized. Research using PISA data, where students were randomly selected and not connected to their teachers directly, emphasized protective factors at the school level (Agasisti et al., 2018). Studies employing TIMSS or PIRLS data, where students were nested within classrooms, provided more detailed information about teachers and their teaching (Garcia-Crespo et al., 2021). Nonetheless, despite these advancements, there remain three methodological issues regarding using ILSAs data to explore academic resilience.

Firstly, very few studies have adequately addressed the issue related to the application of weights. For example, the total student weight (*totowgt*) variable in TIMSS is commonly used in multilevel analysis without proper decomposition, despite its suitability for one-level analyses (Rutkowski et al., 2010). Secondly, scholars exhibit inconsistency in the use of plausible values, with some employing only one plausible value and others using average scores (Garcia-Crespo et al., 2019; Martin et al., 2022), despite the recommendation to use all plausible values to account for measurement error (Mullis and Martin, 2017). Thirdly, ILSAs, such as PISA, often have smaller cluster sizes for disadvantaged students due to sampling 40 students per school. Small cluster sizes may result in reduced statistical power and difficulty detecting significant effects (Anderson et al., 2017).

##### 3.3.2.2 Progressive

Of the 29 studies using progressive operationalization, 22 adopted quantitative methods (see Supplementary Table S2). Most of these studies treated academic resilience as a continuous variable, while seven handled it as a binary outcome. Consequently, linear regression was more commonly used than logistic regression. While the studies in this group focused primarily on individual and family-level protective factors, approximately half also investigated protective factors related to teachers and schools. Despite including school-level factors in some studies during the 2000s, multilevel modeling was not employed (Cappella and Weinstein, 2001; Hawkins and Mulkey, 2005). The use of multilevel modeling has increased for examining multilevel protective factors since the 2010s (Langenkamp, 2010; Wolke et al., 2013), leading to increased attention on interactions between protective factors and the use of weights (Kong, 2020). For example, Langenkamp (2010) utilized national assessment data to explore the impact of protective factors through a stepwise method, incorporating variables from different levels in a progressive manner and taking into account potential interactions. Several scholars analyzing national assessments data have incorporated weights into their analyses (Nichols et al., 2016; Rosen et al., 2019; Kong, 2020).

Explanatory approaches such as cluster analysis and latent class analysis have been used to identify classes/clusters of resilience-promoting factors. Furthermore, Peck et al. (2008) used logistic regression to examine the relationship between academic resilience and identified clusters, in which students were clustered based on their engagement in extra-curricular activities. However, the method assigned a unique class number to each individual rather than using class membership probabilities, failing to account for measurement error. To address this limitation, Asparouhov and Muthén (2014) developed a three-step approach to establish the connection between latent classes, covariates, and distal outcomes while accounting for measurement error. Boutin-Martinez et al. (2019) identified latent classes based on students’ attitudes toward math and their communication with parents, and these classes were linked to covariates (individual demographic variables) and distal outcomes (academic resilience) via three-step method.

##### 3.3.2.3 Instrumental

Out of the 43 studies that employed a scale to measure academic resilience, only one study utilized a mixed-method design, while the remaining relied solely on quantitative approaches (see Supplementary Table S3). Given the treatment of academic resilience as a latent variable in most research, it is not surprising that approximately half of the studies in this group employed structural equation modeling (SEM). Except for one cross-nation study (Granziera et al., 2022), all the other research focused on a specific country. Subsequently, multi-group confirmatory factor analysis (MG-CFA) was utilized to test measurement invariance across various subgroups such as gender, age, and time (Martin and Marsh, 2008; Collie et al., 2015).

About 20% of the studies in this group utilized linear regression to examine the relationship between protective factors and academic resilience, which was operationalized as a score derived from a resilience scale. Several scholars used linear regression with MANOVA, ANOVA, or correlation analysis to examine gender differences or interactions with covariates (Yavuz and Kutlu, 2016; Victor-Aigboidion et al., 2020). Additionally, academic resilience has been less frequently treated as a binary outcome with a threshold applied to resilience scores in research. Salvo-Garrido et al. (2019) employed logistic regression to investigate the relationship between protective factors and academic resilience, utilizing a threshold to define resilient students as those with resilience scores at or below zero.

Cluster analysis and latent profile analysis are also used in studies employing scales to operationalize academic resilience. Putwain and Daly (2013) employed student anxiety and academic resilience to identify clusters and investigate their association with student performance using ANOVA. Similarly, Collie et al. (2017a,b) used items related to support, academic adversity, and academic buoyancy to identify clusters and linked these clusters with students’ motivation through ANOVA. Koirikivi et al. (2021) employed a three-step method to examine the association between latent profiles related to resilience-promoting factors (e.g., peer relationship, self-regulation, caring, school climate) and student wellbeing.

Studies using instrumental operationalization have two distinct characteristics. Firstly, scholars primarily focused on individual protective factors, with only a few investigating multilevel protective factors. Meanwhile, studies utilizing scales to measure academic resilience typically investigate fewer protective factors compared to other operationalization approaches. Secondly, longitudinal designs are more commonly employed in studies utilizing scales to measure academic resilience (Liew et al., 2018; Bostwick et al., 2022). Specifically, nine studies investigated the longitudinal association between academic resilience and protective factors.

## 4 Discussion

### 4.1 Variations in operationalization, data, and research methods

#### 4.1.1 Operationalizations

The three operationalizations of academic resilience—simultaneous, progressive, and instrumental—each carry implications for construct validity and whether they are measuring the same construct (Rudd et al., 2021). The operationalization of academic resilience has a consequential impact on research design and analysis. The simultaneous and progressive operationalizations both involve two core components, risk and positive adaptation, with a focus on the interplay between the individual and the context. However, instrumental operationalization does not distinguish between risk and positive adaptation. The simultaneous operationalization, while useful, limits understanding longitudinal associations. Conversely, the progressive operationalization addresses this limitation by distinguishing risk and positive adaptations over time, thus providing a dynamic perspective on the development of academic resilience. The instrumental operationalization, on the other hand, facilitates examining the interrelationship between protective factors and academic resilience. Although few studies have investigated the similarities and differences among these operationalizations (Rudd et al., 2021), there remains a need for further theoretical and empirical research to comprehensively understand these approaches and their impacts on resilience studies.

#### 4.1.2 Data

Half of the studies examined in this review collected their own data, which offers the flexibility in the choice of measurement tools and can provide unique insights into students’ academic resilience. However, due to the expense of collecting new data, several studies relied on existing national or local assessment data. National assessments have significantly contributed to resilience studies in education, particularly in the United States during the 2000s (Wayman, 2002; Borman and Overman, 2004). Additionally, longitudinal designs have been implemented in several national assessments, including the National Educational Longitudinal Study in the United States and Family Panel Studies in China. These assessments offer a progressive perspective on academic resilience, as they follow students over time and allow for examining changes in protective factors and academic outcomes (Cappella and Weinstein, 2001; Li and Yeung, 2019).

ILSAs provide a global perspective on education systems, allowing comparisons of students’ academic performance across subjects and countries. Consequently, many studies using ILSA data focus on examining academic resilience internationally. Despite its historical roots in homogeneous contexts, academic resilience is increasingly being studied in diverse international contexts, where concerns about its operationalization validity and reliability have emerged (Ye et al., 2021). Moreover, the variability of student participation in different cycles of ILSAs presents challenges for longitudinal studies focusing on individual-level analysis. It is worth noting that several countries have conducted national assessments as an extension of ILSAs, which can be a valuable source for exploring academic resilience across time (Thiessen, 2008).

#### 4.1.3 Research methods

Quantitative methods were predominantly utilized in the reviewed articles, with only 19 studies employing mixed or qualitative approaches. This finding underscores the pervasive reliance on quantitative research methods within the field, while simultaneously highlighting the potential value of incorporating mixed-methods or qualitative designs to obtain a more comprehensive understanding of the research topic.

Quantitative methods in academic resilience research have been characterized by the development of multilevel modeling, which allows for the nesting of students within schools. To this end, several studies have employed various forms of multilevel modeling, with the stepwise approach being the most widely accepted method. However, this method has limitations regarding cross-national comparisons due to the variability of interactions and a limited number of clusters. The former presents challenges in model convergence, while the latter poses challenges in identifying relationships with decreasing statistical power. Several scholars have chosen to investigate the impact of protective factors by utilizing merged ILSAs data, which presents additional challenges, such as neglecting country-specific contexts (Agasisti et al., 2018). Additionally, using ILSAs data brings about further obstacles, such as applying weights and plausible values properly.

The latent class analysis method has emerged as a complement to multilevel modeling in examining academic resilience. For example, including covariances during model identification can aid in accounting for country-specific characteristics. Meanwhile, latent class analysis typically does not significantly reduce the number of clusters, which helps in statistical power. With the development of the three-step method, it is possible to link latent classes to covariates and distal outcomes (Asparouhov and Muthén, 2014). However, this approach is constrained by two methodological issues: class membership shifting and multilevel considerations. While some studies have utilized the three-step method to examine academic resilience (Boutin-Martinez et al., 2019; Koirikivi et al., 2021), further research is necessary to enhance the use of this approach in the field.

### 4.2 Protective factors related to academic resilience

Protective factors at the individual level, such as motivation and engagement, have been well-studied in education. However, protective factors associated with students’ learning processes and practices have received relatively less attention. This disparity can be attributed to the extensive exploration of the former group in psychology. Additionally, measuring learning-related factors may involve complex and context-specific constructs that are difficult to capture with standardized measures. For example, most studies examining the learning process are based on self-collected data (Yavuz and Kutlu, 2016) rather than national assessments or ILSAs data.

Although individual factors are extensively researched across studies, patterns emerge considering operationalizations. Specifically, the “instrumentals” approach primarily focuses on individual factors, while the other two approaches give them comparatively less attention.

In education, the emphasis on school-level protective factors is more prominent compared to resilience studies in psychology and sociology. Recognizing that individual or family factors are less likely to be impacted by educational policies, there has been a growing interest in identifying school-level protective factors, particularly those considered malleable, such as school resources. Specifically, using ILSAs data, such as TIMSS and PIRLS, has shed light on areas that were less studied before, such as instructional quality (Granziera et al., 2022).

The investigation of family, peer, and community factors in academic resilience research is limited. However, it is noteworthy that connecting cross-sectional data with those from government agency or public organizations offer valuable insights into the associations between students’ academic performance and their familial, social, and community contexts (Fantuzzo et al., 2012).

It is imperative to underscore that protective factors serve not merely as catalysts for academic resilience, fostering enhanced academic outcomes in both cognitive and non-cognitive areas, but they may also represent a form of positive adaptation. For instance, increased motivation can drive disadvantaged students to achieve better academically, which in turn can boost their motivation even further. Although some researchers, particularly those using the instrumental approaches (e.g., Marsh et al., 2013), have investigated the reciprocal relationship between protective factors and academic resilience, this interplay receives limited attention in the field, especially in studies that do not use longitudinal data.

### 4.3 Limitations and future directions

The scope of this study is restricted to papers containing the search terms in the title, which may result in the omission of pertinent studies that were not identified. Some studies meeting the inclusion criteria of this review may focus on how disadvantaged students, such as minorities, cope with adversity and perform well instead of directly examining academic resilience. To minimize the potential confounding effects of intervention programs, this review deliberately excludes studies that evaluate such interventions, which may have led to the unintentional omission of some protective factors associated with the community.

Future research using cross-section data should consider incorporating the local context and treating academic resilience as dynamic process, which can provide additional insights into the impact of protective factors. One potential approach is to identify resilient students using ILSAs data and then observe teaching practices in classrooms with a high proportion of such students. To account for the variation across groups, it is advisable to consider the hierarchical structure when investigating the influence of protective factors, even those within the same level. Additionally, given the interdisciplinary nature of resilience research, adopting statistical methods developed in other fields could be beneficial, though such integration requires careful consideration.

This study employed a systematic review approach, which, despite offering a comprehensive overview of existing literature, is limited in its ability to quantify variations and impacts across studies due to its qualitative nature. Future research would benefit from employing meta-analysis to thoroughly examine the heterogeneity index and effect sizes across studies in academic resilience. This method would facilitate a detailed quantitative synthesis, enhancing understanding of variations and impacts across studies and improving the consistency and reliability of findings.

## 5 Conclusion

This systematic review offers valuable insights into the protective factors associated with academic resilience, highlighting their crucial role in facilitating the success of disadvantaged students. By examining the relationships between academic resilience and five groups of protective factors across individual, school, family, peer, and community, this review offers a comprehensive overview of the current state of research in the field. The present study further explores the relationships between protective factors, their operationalizations, data sources, and research methods employed. Notably, individual factors have received extensive investigation, often using instrumental operationalization and utilizing structural equation modeling. School-level protective factors, especially those related to teaching, have gained increasing attention with the development of ILSAs. The use of multilevel modeling approach has also gained prominence in exploring the influence of school-level factors. These findings have important implications for the development of targeted interventions, resource allocation, and evidence-based strategies aimed at fostering academic resilience. Ultimately, by addressing the needs of disadvantaged students, these efforts strive to promote greater equity and improve educational outcomes for all.

## Data Availability

The original contributions presented in the study are included in the article/Supplementary material, further inquiries can be directed to the corresponding author.
