# Supplementary material for: Systematic review of protective factors related to academic resilience in children and adolescents: unpacking the interplay of operationalization, data, and research method
Source: Front Psychol. 2024 Aug 21;15:1405786. doi: 10.3389/fpsyg.2024.1405786 (PMC11371752; doi:10.3389/fpsyg.2024.1405786)
Supplement: Supplementary file 1 [file Data_Sheet_1.docx]

**Table A1** Studies with a “simultaneous” operationalization (*n* = 44)

| **First Author** | **Year** | **Country** | **Risk** | **Positive Adaptation** | **Main Methods** | **Sample** | **Data** |
| --- | --- | --- | --- | --- | --- | --- | --- |
| Borman | 2004 | United States | low-SES | mathematics performance | MANOVA | 3981 students (grade 3) | Prospects: The Congressionally Mandated Study of Educational Growth and Opportunity |
| Gayles | 2005 | United States | African American; from a non-affluent school and a non-affluent home | top 10% performance | Open-ended interview; Narrative analysis | 3 high school seniors |  |
| Plunkett | 2008 | United States | Mexican-origin adolescents | academic success (academic motivation, academic satisfaction, Grade Point Average) | MANOVA; Correlation; Dominance analysis | 216 Mexican-origin adolescents living in two-parent intact families |  |
| Gizir | 2009 | Turkey | low-SES | academic performance | Structural equation modeling | 872 students (grade 8) |  |
| Cunningham | 2010 | United States | low-SES | academic and general success | Hierarchical linear regressions | 206 African American high school students |  |
| Tinsley | 2010 | United States | low resource youth | educational expectations | Hierarchical linear regressions | 697 high school students |  |
| Rana | 2011 | United States | unaccompanied refugee minors | educational attainment | Open-ended semi-structured interviews | 19 Sudanese refugees and 20 parents from 15 families |  |
| Agasisti | 2012 | Italy | low-SES | school achievement | Multilevel logistic regression | PISA 2009 Italy |  |
| Fantuzzo | 2012 | United States | risk score based on child maltreatment, maternal education at birth, homeless shelter stay, prenatal care, preterm/low birth weight, and lead exposure | academic success | Multilevel linear regression models | 2930 African American boys and 605 White boys | The Pennsylvania System of School Assessment (PSSA); TerraNova (CTB/McGraw-Hill, 1997, 2001) |
| Rivera | 2012 | United States | living in economically and socially disadvantaged circumstances | successful in school | Classroom observation; ANOVA | 189 students (grades 4 & 5) |  |
| Cheung | 2014 | Shanghai, Hong Kong, Korea, Singapore | low-SES | academic performance | Logistic regression | PISA 2009 data from Shanghai, Korea, Hong Kong, and Singapore | PISA 2009 |
| Downey | 2014 | United States | serious life difficulties | academic performance | Constructed situation interview | 50 children (ages 8-12) |  |
| Graves | 2014 | United States | race/ethnicity | academic performance | Interview | 4 students (grade 10) |  |
| Agasisti | 2014a | OECD | low-SES | academic performance | Multilevel logistic regression | PISA 2009 OECD | PISA 2009 |
| Agasisti | 2014b | OECD | low-SES | academic performance | Fixed Effects models | PISA OECD 2000-2012 | PISA 2000-2012 |
| Erberber | 2015 | 28 Education Systems | low-SES | academic performance | Logistic regression analysis | TIMSS 2011 (grade 8) 28 Education Systems | TIMSS 2011 |
| Kosciw | 2015 | United States | lesbian, gay, bisexual, and transgender (LGBT) youth | well-being, academic performance (GPA, missed school days) | Multi-group structural equation modeling | 7816 LGBT secondary school students |  |
| Rojas Flórez | 2015 | Colombia | from a school that is located in a low-income and marginalized area of the city | academic performance | Case study; Document analysis; Interview with students, teachers, and parents | 6 students |  |
| Anagnostaki | 2016 | Greece | social risks and immigrant status | academic performance (average grades on five main subjects) | Hierarchical regression analysis | 300 middle school adolescents |  |
| Sandoval-Hernandez | 2016 | Singapore, Korea, Hong Kong, Chinese Taipei | low-SES | academic performance | Logistic regression | TIMSS 2011 data from Singapore, Korea, Hong Kong, Chinese Taipei | TIMSS 2011 |
| Wittrup | 2016 | United States | school-based discrimination | academic engagement | Hierarchical regressions | 663 African American adolescents (ages 12-19) |  |
| Celik | 2017 | Turkey | from disadvantaged neighborhoods | academic performance | Semi-structured in-depth interviews | 13 resilient student-mother pairs and 10 dropout-mother pairs |  |
| Cheung | 2017 | Shanghai, Singapore, Hong Kong, Chinese Taipei, and Korea | low-SES | academic performance | Logistic regression | PISA 2012 data from Shanghai, Singapore, Hong Kong, Chinese Taipei, and Korea | PISA 2012 |
| Li | 2017 | China | competitive academic environments | academic performance | Confirmatory factor analysis; Structural equation modeling | 693 students (grade 11) |  |
| Agasisti | 2018 | PISA participants | low-SES | academic performance | Multilevel logistic analysis | PISA cycles (2006-2015) | PISA cycles (2006-2015) |
| Garcia-Crespo | 2019 | EU | low-SES | academic performance | Hierarchical logistic analysis | PIRLS 2016 (23 EU countries) | PIRLS 2016 |
| Li | 2019 | China | rural student | academic performance | Correlation; Multiple regressions | 1212 children (ages 10–15) | Chinese Family Panel Studies (2012) |
| Stevenson | 2019 | United States | Latino | success in STEM fields | Semi-structured interviews | 3 Latina high school students |  |
| Salvo-Garrido | 2019 | Chile | Low-SES | language achievement | Multilevel logistic regression | SIMCE Language test grade 4 | Sistema de Medició de Calidad de la Educación (SIMCE) |
| Hofmeyr | 2019 | South Africa | low-SES | academic performance | Multilvel logistic regression | PIRLS 2016 and TIMSS 2015 in South Africa | PIRLS 2016; TIMSS 2015 |
| De Feyter | 2020 | United States | from low-income, immigrant families | academic performance | Multivariate and logistic regression analyses (cluster considered) | 1638 low-income Latino and Black children | Miami School Readiness Project (MSRP) |
| Ge | 2020 | China | poverty | academic performance | Multilevel structural equation modeling | 1132 children (ages 10-15) | Chinese Family Panel Study (CFPS:2014) |
| Wood | 2020 | Canada | Inuit youth | managing lives well (identified by mental health staff) | Image-based qualitative elicitation methods, thematic analysis | 8 youth (ages 13–17) |  |
| Garcia-Crespo | 2021 | EU | low-SES | reading performance | Multilevel logistic regression models | PIRLS 2016 EU | PIRLS 2016 |
| Kothari | 2021 | United States | foster care | three educationally resilient outcomes (math, reading, and attendance) | Logistic regressions | 315 youth in foster care (grades 1-10) | Supporting Siblings in Foster Care Study (SIBS-FC ) |
| Vicente | 2021 | OECD | low-SES | academic performance | Multilevel probit analysis | PISA OECD (2003-2018) | PISA (2003-2018) |
| Süleyman | 2022 | Turkey | low-SES | academic performance | Binary logistic regression analysis | PISA Turkey 2018 | PISA 2018 |
| Cui | 2022 | China | low-SES | academic performance | Multilevel logistic regression models | 1767 eighth-graders | Regional Education Monitoring Project (REMP) |
| Gabrielli | 2022 | Greece, Italy, and Spain; France, Germany, the Netherlands, and the United Kingdom | low-SES | an adequate level of proficiency in reading, science, and math | Two-level logistic random intercept models | PISA 2015 AND 2018 from 7 EU countries | PISA 2015 & 2018 |
| Garcia-Crespo | 2022 | EU | low-SES | academic performance | Three-level logistic regression models | TIMSS 2019 EU | TIMSS 2019 |
| Jin | 2022 | China | CART classification tree | CART classification tree | Classification and Regression Tree and Multilevel Logistic Regression modeling | PISA 2015 B-S-J-G (China) | PISA 2015 |
| Martin | 2022 | 18 EU countries | low-SES | academic performance | Multilevel probit regression | PISA 2015 EU | PISA 2015 |
| Özcan | 2022 | 64 education systems | low-SES | academic performance | Multi-group logistic regression analyses (cluster considered) | PISA 2018 | PISA 2018 |
| Jang | 2023 | United States | low-SES | reading performance | Multi-group structural equation model analysis | PISA 2018 US | PISA 2018 |

**Table A2** Studies with a “progressive” operationalization (*n* = 29) and studies without a clear operationalization (*n* = 3)

| **First Author** | **Year** | **Country** | **Risk** | **Positive Adaptation** | **Main Methods** | **Sample** | **Data** |
| --- | --- | --- | --- | --- | --- | --- | --- |
| Cappella | 2001 | United States | Low proficiency in reading in 8th grade | significant positive change in 12th grade | Regression analyses; Path analysis | 1362 students (grades 8-12) | National Educational Longitudinal Study of 1988 (NELS-88) |
| Wayman | 2002 | United States | dropouts | degree attainment | Logistic regression | 1071 dropouts (519 followed up) | National Institute on Drug Abuse |
| Crosnoe | 2004 | United States | problematic relationships with parents | academic behavior | Structural models | 11788 adolescents (grades 7-12) | National Longitudinal Study of Adolescent Health |
| Randolph | 2004 | United States | low-income, single-parent families | high school completion | Survivor functions and Cox event history modeling; multivariate analysis | 692 youth |  |
| Schoon | 2004 | United Kingdom | low-SES | adult attainments at age 33 | Linear Regression, path analysis, multi-group analysis | 9716 16-year-olds | National Child Development Study (NCDS) |
| Hawkins | 2005 | United States | African American | complete school, attend university | Multiple regression analyses | Male 1105 Female 1112 (grade 8) | National Education Longitudinal Study of 1988 (NELS, 99) |
| Sacker | 2007 | United Kingdom | low-SES | return to full-time education | Logistic regression | 12940 students | National Child Development Study (NCDS) |
| Morales | 2008 | United States | low-SES and ethnic minority status | academic performance | Ethnographic semi-structured interviews; Email and phone call follow-up | 5 students |  |
| Peck | 2008 | United States | vulnerable youth characterized by significant risks and an absence of assets (accessed at age 14) | college attendance | Cluster analysis; logistic regression | Time 1: 1060 students (age 14); Time 2: 1057 students (age 17); Time 3: 912 students (age 19); Time 4: 887 students (age 21) | Maryland Adolescent Development in Context Study (MADICS) |
| Thiessen | 2008 | Canada | low performance in reading at age 15 | completing high school, participating in post-secondary education | ANOVA; Multinomial logistic regression | 6342 students (ages 15-19) | Canadian Longitudinal Youth in Transition Survey (YITS), an extension of PISA 2000 |
| Gastic | 2009 | United States | sexual minority youth | educational success (post-secondary participation) | Logistic regression models | 4882 students | National Longitudinal Study of Adolescent Health |
| Kvalsund | 2010 | Norway | special need students | social inclusion | Logistic regression | around 500 students (from upper secondary school to age 22) | Data from 2 National Studies: Reform 94-Students with Special Educational Needs; Adult Life on Special Terms |
| Langenkamp | 2010 | United States | struggle academically | not drop out | MANOVA; hierarchical linear modeling | 2065 students | National Longitudinal Study of Adolescent Health; Adolescent Health and Academic Achievement (AHAA) study. |
| Pan | 2011 | Chinese Taipei | low-income family | academic performance | interviews followed up two years later | 60 family dyads | Taiwan Youth Project |
| Wolke | 2013 | Germany | preterm/low birth weight | school success at age 13 | Hierarchical regression model | 314 very preterm/very low birth weight and 338 term control children | Bavarian Longitudinal Study |
| Paat | 2015 | United States | Mexican immigrant children | academic achievement | Multiple regression analyses | Time 1: 755 high school students of Mexican origin (ages 13-17); Time 2: 3 years later response rate 81.5%; Time 3: 10 years after Time 1 response rate 68.9% | The Children of Immigrants Longitudinal Study (CILS) |
| Nichols | 2016 | United States | with incarcerated parents | educational attainment (truancy; academic achievement; and lifetime educational attainment) | Multilevel linear models | Datasets: Truancy: 71447 students; Highest Level of Education: 69082 students; Cumulative Academic Achievement: 46045 students | National Longitudinal Survey of Adolescent Health (Wave I ages 12-18) and Wave IV ages 24-32); Academic Achievement dataset (AHAA) |
| Strolin-Goltzman | 2016 | United States | former foster youth | college entry or intent to attend college | Survey; interview; logistic regression analysis | Survey: 102 youth (ages 15-21); Interview: 10 youth (age 18-22) |  |
| Neal | 2017 | United States | former foster students | matriculated to a highly selective institution | survey, interview | Survey 57 students; Interview 11 students |  |
| Watson | 2017 | United States | teen mother | graduate high school | Case study; personal interviews | 6 parenting teen mothers |  |
| Boutin-Martinez | 2019 | United States | Latina/o high school students | Grade 12 mathematics achievement, dropout rates, and enrollment in post-secondary education | Latent class analysis (three-step) | 1610 high school Latina/o students | Education Longitudinal Study of 2002 (ELS: 2002) |
| Rosen | 2019 | United States | extended school absences or dropout episodes | return to complete a diploma or GED within 4 years of starting high school | Multinomial logistic regression | 2320 students who had experienced a dropout episode | High School Longitudinal Study of 2009 (HSLS:09) |
| Wills | 2019 | South Africa | Poverty | perform above socio-economic expectations in literacy | Logistic regression models | 2656 students for pre-test (beginning of school year grade 6) 2383 for post-test (end of school year grade 6) | “Leadership for Literacy” Project |
| Bussemakers | 2020 | the Netherlands | youth adversity (between 5 and 12 years old) | educational attainment (highest degree) | Ordinary Least Squares regression | 5760 respondents | Family Survey Dutch Population (FSDP) conducted in 2000, 2003 and 2009 |
| Kong | 2020 | Ireland | low-SES (age 9) | academic achievement (age 13) | Multilevel regression analyses | about 7000 children | Growing Up in Ireland (GUI) Longitudinal Study (Wave 1 & Wave 2, Child Cohort) |
| Kumi-Yeboah | 2020 | United States. | immigrant youth | educational success | Interview (twice in 2 years) | 60 high school students |  |
| Corwith | 2022 | South Sudan | Teenage Mothers | return to school and continue education | Semi-structured  interview | 49 youth (ages 16-19) |  |
| Jaramillo | 2022 | United States | youth in foster care | high school completion | Multinomial logistic regression | 208 foster youth (ages 16.5-18.5) | My Life Mentoring program |
| Williams | 2022 | Australia | begin school with poorer developmental competencies | academic performance (Grade 3) | MANOVA | 2.118 Children (from entry to grade 3) | Growing Up in Australia: The Longitudinal Study of Australian Children (LSAC) |
| Oldfield | 2020 | Guatemala | not specified | not specified | ethnographic interviews and image elicitation | 8 adolescents (ages 11-16) and 4 project coordinators |  |
| Austin | 2022 | United States | not specified | not specified | Structured interviews, surveys, Multiple linear regression analyses | 75 students (ages 8-14) |  |
| Fenwick | 2022 | Ireland | not specified | not specified | Pearson correlations | 405 students attending 16 DEIS (Delivering Equality of Opportunity in Schools) | The Power 2 Progress (P2P) intervention; |

**Table A3** Studies with an “instrumental” operationalization (*n* = 43)

| **First Author** | | **Year** | | **Country** | | **Scale** | | **Main Methods** | | **Sample** | | **Data** |
| --- | --- | --- | --- | --- | --- | --- | --- | --- | --- | --- | --- | --- |
| Nota | | 2004 | | Italy | | scale (not specified) | | Survey; interview; MANOVA; Regression analysis | | Time 1: 81 high school students; Time 2: 49 students (3 years later) | |  |
| Martin | | 2008 | | Australia | | 4-item (Martin, 2006) | | Multi-group confirmatory factor analysis; structural equation modeling (longitudinal process model) | | 598 students (grades 8 &10); Time 1: halfway through the school year Time 2: at the end of the same school year | |  |
| Martin | | 2010 | | Australia | | Academic Buoyancy Scale (ABS; Martin & Marsh, 2008) | | Multi-group confirmatory analysis and structural equation modeling; Linear regression analysis | | 1866 high school students (Time 2: one year after Time 1) | |  |
| Schelble | | 2010 | | United States | | The subscale of the Child and Adolescent Functional Assessment Scale (CAFAS; Hodges & Wong, 1996) | | Linear regression analysis | | 158 maltreated children | |  |
| Martin | | 2013 | | Australia | | Academic Buoyancy Scale (ABS; Martin & Marsh, 2008) | | Cross-lagged structural equation modeling | | 2971 students (Time 2: one year after Time 1) | |  |
| Miller | | 2013 | | United Kingdom | | Academic Buoyancy Scale (ABS; Martin & Marsh, 2008) | | Ordinal logistic regression models | | 1081 students (ages 7-11) | |  |
| Putwain | | 2013 | | United Kingdom | | Academic Buoyancy Scale (ABS; Martin & Marsh, 2008) | | Cluster analysis | | 469 secondary school students | |  |
| Skinner | | 2013 | | United States | | 3 subscales: engagement, emotional reactivity, and re-engagement (Skinner & Pitzer, 2012) | | Confirmatory factor analysis; Structural equation modeling | | 1020 students (grades 3 & 6); Time 1& 2: spring and fall in the same school year | |  |
| Wu | | 2014 | | China | | Connor-Davidson Resilience Scale (CD-RISC; Connor and Davidson, 2003) | | Structural equation modeling | | 806 migrant children (grades 7-9) | |  |
| Yu | | 2014 | | China | | Academic Buoyancy Scale (ABS; Martin & Marsh, 2008) | | Confirmatory factor analysis; Hierarchical path analysis | | 3753 students (grades 7-9) | |  |
| Collie | | 2015 | | Australia | | Academic Buoyancy Scale (ABS; Martin & Marsh, 2008) | | Confirmatory factor analysis and structural equation modeling | | 2971 high school students (Time 2: one year after Time 1) | |  |
| Yavuz | | 2016 | | Turkey | | Resilience Scale for Adults (Friborg, Hjemdal, Rosenvinge & Martinussen, 2003) | | Regression Analysis; MANOVA | | 304 High school seniors | |  |
| Bakhshaee | | 2017 | | Iran | | Dehghanizadeh and Husseinchari’s Questionnaire (2012) | | Structural equation modeling | | 400 female high school students | |  |
| Collie | | 2017 | | Australia | | 10 risk items from the Academic Risk and Resilience Scale (Martin, 2013) | | Cluster analysis; ANOVA and SNK (Student-Newman-Keuls) post hoc tests | | 249 young people (age 16-20) | |  |
| Liew | | 2018 | | United States | | California Child Q-Set (Caspi, Block, Block, & Klopp, 1992) | | longitudinal mediation models (cluster considered) | | 784 students (around age 7), Time 1: Year 1; Time 2: Year 2; Time 3: Year 3 | |  |
| Bellis | | 2018 | | United Kingdom | | Child and Youth Resilience Measure (CYRM-12; Liebenberg, Ungar & LeBlanc, 2013 ) | | Logistic regression | | 2452 interviewees (age 18-69) with Adverse childhood experiences (ACEs) before 18 | | National (Wales) cross-sectional retrospective survey |
| Chen | | 2018 | | China | | Ego-resiliency scale (Block & Kremen, 1996) | | Regression analyses | | 484 secondary school students (grade 10) | |  |
| Trigueros | | 2019 | | Spain | | Scale on Resilience in PE classes (developed by authors) | | Structural equations model | | 615 secondary school students (ages 14-19) | |  |
| Yu | | 2019 | | China | | Academic Buoyancy Scale (ABS; Martin & Marsh, 2008) | | Confirmatory factor analysis; hierarchical linear regressions | | 2434 middle school students (ages 11-15) | |  |
| Rachmawati | | 2020 | | Indonesia | | scale (not specified) | | ANOVA; regression analysis | | 315 middle school students (grade 7) | |  |
| Victor-Aigboidion | | 2020 | | Nigeria | | Academic Risk and Resilience Scale (ARRS; Martin, 2013) | | Pearson correlation; regression analysis | | 1320 junior secondary two (SSII) students | |  |
| Kheirkhah | | 2020 | | Iran | | Academic Resilience Inventory (ARI: Samouels, 2004) | | MANOVA; ANOVA | | 30 students (first-grade high school) | |  |
| Bester | | 2020 | | South Africa | | 36 items  6-point Likert scale (developed by authors) | | Correlation; Regression analysis | | 117 students (grades 9-10) | |  |
| Fang | | 2020 | | China | | three items (4-point Likert scale) from Chinese Education Panel Survey (CEPS) | | Partial Least Squares Structural Equation Modeling | | 2328 children (ages 13-15) | | Chinese Education Panel Survey (CEPS) (2013-2014) |
| Fiorilli | | 2020 | | Italy | | Questionnaire for Anxiety and Resilience (De Beni et al., 2014) | | Structural equation modeling | | 1235 high school students (ages 13-17) | |  |
| Liu | | 2020 | | China | | 9-item scale (Ricketts, Engelhard, and Chang, 2015) | | Confirmatory factor analysis; structural equation modeling | | 751 students (grades 9-10) | |  |
| Yustika | | 2021 | | Indonesia | | Academic Resilience Scale-30 (ARS-30; Cassidy, 2016) | | Correlation | | 277 high school students (grade 12) | |  |
| Mohan | | 2021 | | India | | Motivation and engagement Scale High-School (MES-HS; 12th edition; Martin, 2002) | | Correlation , t-test | | 120 students (ages 13-18) | |  |
| af Ursin | | 2021 | | Finland | | Academic Buoyancy Scale (ABS; Martin & Marsh, 2008) | | Confirmatory factor analysis; structural equation modeling | | 403 children (ages 8-9 ) | |  |
| Armfield | | 2021 | | Australia | | Multiple Strength Indicator (MSI) of the Early Australian Development Census (AEDC) | | Logistic regression analysis (univariate and multivariate models) | | 3414 high-risk children with a resilience indicator | | Early Australian Development Census (AEDC) |
| Baniani | | 2021 | | Iran | | Academic Resilience Inventory (ARI: Samouels, 2004) | | Regression analysis | | 162 high school students | |  |
| Kim | | 2021 | | Korea | | six-item scale (Kapikiran, 2012) | | Autoregressive cross-lagged modeling | | about 900 Elementary and middle school students were studied for three years (2015-2017) | | Education Welfare Priority Project |
| Koirikivi | | 2021 | | Finland | | 4 subdimensions scale (Ungar & Liebenberg, 2011) | | Latent profile analysis (three-step) | | 2837 respondents (ages 16-19) | |  |
| Lei | | 2021 | | China | | Academic Buoyancy Scale (ABS; Martin & Marsh, 2008) | | Structural equation modeling | | 560 high school students | |  |
| Chitra | | 2022 | | India | | Academic Resilience Scale (developed by authors) | | ANOVA; regression analysis | | 200 secondary school students | |  |
| Adigun | | 2022 | | South Africa | | The Academic Resilience Scale (ARS-30; Cassidy, 2016) | | Confirmatory factor analysis; structural equation modeling | | 292 deaf learners (grades 8-10) | |  |
| Bostwick | | 2022 | | Australia | | Academic Buoyancy Scale (ABS; Martin & Marsh, 2008) | | single-level (students) and doubly-latent multilevel (students and schools) cross-lagged structural equation models | | 71681 students (grades 7-11) | | “Tell Them From Me” (TTFM) student survey |
| Chen | | 2022 | | China | | 14-item Ego- Resilience Scale (Block & Kremen, 1996) | | Multiple Regressions | | 635 students (ages 14-19) | |  |
| Dong | | 2022 | | Iran | | Academic Resilience Inventory (ARI: Samouels, 2004) | | ANOVA | | 300 third-grade high school students | |  |
| Granziera | | 2022 | | Singapore and Australia | | Academic Buoyancy Scale (ABS; Martin & Marsh, 2008) | | Structural equation modeling, Path analysis, regression analysis | | Study 1: 2510 grade 9 students from Singapore (cross-sectional); Study 2: 119 elementary school students from Australia (Time 2: 6-9 months after Time 1) | |  |
| Shao | | 2022 | | China | | five-item (Cassidy, 2016) | | Confirmatory factor analysis; structural equation modeling | | 250 middle school students | |  |
| Trigueros | | 2022 | | Spain | | Resilience Scale (developed by authors) | | Structural equation modeling | | 2856 participants (Mean age =14.31) | |  |
| Zaw | | 2022 | | Thailand | | 29-item Grotberg’s resilience scale (Grotberg, 1995) | | multivariate logistic regression analysis | | 6167 adolescents (ages 13-18) | | 2019 Bangkok Behavioral Surveillance Survey (BBSS) |
