# Supplementary material for: Systematic review of protective factors related to academic resilience in children and adolescents: unpacking the interplay of operationalization, data, and research method
Source: Front Psychol. 2024 Aug 21;15:1405786. doi: 10.3389/fpsyg.2024.1405786 (PMC11371752; doi:10.3389/fpsyg.2024.1405786)
Supplement: Supplementary file 2 [file Table_1.docx]

# References for Supplementary Materials

Aburn, G., Gott, M., & Hoare, K. (2016). What is resilience? An integrative review of the empirical literature. *Journal of advanced nursing, 72*(5), 980-1000. https://doi.org/10.1111/jan.12888

Adigun, O. T., & Ndwandwe, N. D. (2022). Academic Resilience Among Deaf Learners During E-Learning in the COVID-19 Era. *Research in Social Sciences and Technology*, *7*(2), 27-48. <https://doi.org/10.46303/ressat.2022.8>

af Ursin, P., Järvinen, T., & Pihlaja, P. (2021). The Role of Academic Buoyancy and Social Support in Mediating Associations between Academic Stress and School Engagement in Finnish Primary School Children. *Scandinavian Journal of Educational Research, 65*(4), 661-675. https://doi.org/10.1080/00313831.2020.1739135

Agasisti, T., Avvisati, F., Borgonovi, F., & Longobardi, S. (2018). Academic resilience: What schools and countries do to help advantaged students succeed in PISA. *OECD Education Working Papers, 167*. https://doi.org/10.1787/e22490ac-en

Agasisti, T., & Longobardi, S. (2012). Inequality in education: can Italian disadvantaged students close the gap? A focus on resilience in the Italian school system. *Documents de treball IEB*(39), 1-46. http://dx.doi.org/10.1016/j.socec.2014.05.002

Agasisti, T., & Longobardi, S. (2014a). Educational institutions, resources, and students' resiliency: an empirical study about OECD countries. *Economics Bulletin, 34*(2), 1055-1067.

Agasisti, T., Longobardi, S., & Regoli, A. (2014b). Does public spending improve educational resilience? *A longitudinal analysis of OECD-PISA data* (No. 3).

Anagnostaki, L., Pavlopoulos, V., Obradovic, J., Masten, A., & Motti-Stefanidi, F. (2016). Academic resilience of immigrant youth in Greek schools: Personal and family resources. *European Journal of Developmental Psychology, 13*(3), 377-393. https://dx.doi.org/10.1080/17405629.2016.1168738

Armfield, J. M., Ey, L. A., Zufferey, C., Gnanamanickam, E. S., & Segal, L. (2021). Educational strengths and functional resilience at the start of primary school following child maltreatment. *Child Abuse & Neglect, 122*. https://dx.doi.org/10.1016/j.chiabu.2021.105301

Asparouhov, T., & Muthén, B. (2014). Auxiliary variables in mixture modeling: Three-step approaches using Mplus. *Structural Equation Modeling: A Multidisciplinary Journal, 21*(3), 329-341. https://doi.org/10.1080/10705511.2014.915181

Austin, J. L., Jeffries, E. F., Winston, W., & Brady, S. S. (2022). Race-related Stressors and Resources for Resilience: Associations With Emotional Health, Conduct Problems, and Academic Investment Among African American Early Adolescents. *Journal of the American Academy of Child and Adolescent Psychiatry, 61*(4), 544-553. https://doi.org/10.1016/j.jaac.2021.05.020

Bakhshaee, F., Hejazi, E., Dortaj, F., & Farzad, V. (2017). Self-management strategies of life, positive youth development and academic buoyancy: A causal model. *International Journal of Mental Health and Addiction, 15*(2), 339-349. https://dx.doi.org/10.1007/s11469-016-9707-x

Baniani, P., & Davoodi, A. (2021). Predicting Academic Resilience based on Metacognitive Beliefs and Achievement Motivation in High School Students in Shiraz, Iran. *International Journal of Pediatrics, 9*(6), 13765-13772. https://doi.org/10.22038/ijp.2020.53686.4257

Bellis, M. A., Hughes, K., Ford, K., Hardcastle, K. A., Sharp, C. A., Wood, S., . . . Davies, A. (2018). Adverse childhood experiences and sources of childhood resilience: a retrospective study of their combined relationships with child health and educational attendance. *BMC Public Health, 18*(1), 1-12. https://doi.org/10.1186/s12889-018-5699-8

Bester, G., & Kuyper, N. (2020). The Influence of Additional Educational Support on Poverty-Stricken Adolescents' Resilience and Academic Performance. *Africa Education Review, 17*(3), 158-174. http://dx.doi.org/10.1080/18146627.2019.1689149

Block, J., & Kremen, A. M. (1996). IQ and ego-resiliency: conceptual and empirical connections and separateness. *Journal of Personality and Social Psychology*, *70*(2), 349. https://doi.org/10.1037/0022-3514.70.2.349

Borman, G. D., & Overman, L. T. (2004). Academic resilience in mathematics among poor and minority students. *The Elementary School Journal, 104*(3), 177-195.

Bostwick, K. C. P., Martin, A. J., Collie, R. J., Burns, E. C., Hare, N., Cox, S., . . . McCarthy, I. (2022). Academic buoyancy in high school: A cross-lagged multilevel modeling approach exploring reciprocal effects with perceived school support, motivation, and engagement. *Journal of Educational Psychology*. https://dx.doi.org/10.1037/edu0000753

Boutin-Martinez, A., Mireles-Rios, R., Nylund-Gibson, K., & Simon, O. (2019). Exploring Resilience in Latina/o Academic Outcomes: A Latent Class Approach. *Journal of Education for Students Placed at Risk, 24*(2), 174-191. http://dx.doi.org/10.1080/10824669.2019.1594817

Bussemakers, C., & Kraaykamp, G. (2020). Youth adversity, parental resources and educational attainment: Contrasting a resilience and a reproduction perspective. *Research in Social Stratification and Mobility, 67*. http://dx.doi.org/10.1016/j.rssm.2020.100505

Cappella, E., & Weinstein, R. S. (2001). Turning around reading achievement: Predictors of high school students' academic resilience. *Journal of Educational Psychology, 93*(4), 758. https://doi.org/10.1037/0022-0663.93.4.758

Cassidy, S. (2016). The Academic Resilience Scale (ARS-30): A new multidimensional construct measure. *Frontiers in psychology*, *7*, 1787. https://doi.org/10.3389/fpsyg.2016.01787

Caspi, A., Block, J., Block, J. H., Klopp, B., Lynam, D., Moffitt, T. E., & Stouthamer-Loeber, M. (1992). A" common-language" version of the California Child Q-Set for personality assessment. *Psychological Assessment*, *4*(4), 512. https://doi.org/10.1037/1040-3590.4.4.512

Çelik, Ç. (2017). Parental networks, ethnicity, and social and cultural capital: The societal dynamics of educational resilience in Turkey. *British Journal of Sociology of Education, 38*(7), 1007-1021. https://doi.org/10.1080/01425692.2016.1218753

Chen, X. J., Cheung, H. Y., Fan, X. T., & Wu, J. (2018). Factors related to resilience of academically gifted students in the Chinese cultural and educational environment. *Psychology in the Schools, 55*(2), 107-119. https://doi.org/10.1002/pits.22044

Chen, X. J., & Padilla, A. M. (2022). Emotions and creativity as predictors of resilience among L3 learners in the Chinese educational context. *Current Psychology, 41*(1), 406-416. https://doi.org/10.1007/s12144-019-00581-7

Cheung, K. C. (2017). The Effects of Resilience in Learning Variables on Mathematical Literacy Performance: A Study of Learning Characteristics of the Academic Resilient and Advantaged Low Achievers in Shanghai, Singapore, Hong Kong, Taiwan and Korea. *Educational Psychology, 37*(8), 965-982. https://doi.org/10.1080/01443410.2016.1194372

Cheung, K. C., Sit, P. S., Soh, K. C., Ieong, M. K., & Mak, S. K. (2014). Predicting Academic Resilience with Reading Engagement and Demographic Variables: Comparing Shanghai, Hong Kong, Korea, and Singapore from the PISA Perspective. *The Asia-Pacific Education Researcher, 23*(4), 895-909. https://doi.org/10.1007/s40299-013-0143-4

Chitra, L., & Binuraj, A. (2022). Predictive Efficiency Of Self Efficacy On Academic Resilience Of Secondary School Students. *Journal of Positive School Psychology*, *6*(8), 810-822.

Collie, R. J., Ginns, P., Martin, A. J., & Papworth, B. (2017). Academic buoyancy mediates academic anxiety’s effects on learning strategies: an investigation of English- and Chinese-speaking Australian students. *Educational Psychology, 37*(8), 947-964. https://doi.org/10.1080/01443410.2017.1291910

Collie, R. J., Martin, A. J., Bottrell, D., Armstrong, D., Ungar, M., & Liebenberg, L. (2017). Social Support, Academic Adversity and Academic Buoyancy: A Person-Centred Analysis and Implications for Academic Outcomes. *Educational Psychology, 37*(5), 550-564. http://dx.doi.org/10.1080/01443410.2015.1127330

Collie, R. J., Martin, A. J., Malmberg, L. E., Hall, J., & Ginns, P. (2015). Academic Buoyancy, Student's Achievement, and the Linking Role of Control: A Cross-Lagged Analysis of High School Students. *British Journal of Educational Psychology, 85*(1), 113-130. http://dx.doi.org/10.1111/bjep.12066

Connor, K. M., & Davidson, J. R. (2003). Development of a new resilience scale: The Connor‐Davidson resilience scale (CD‐RISC). *Depression and anxiety*, *18*(2), 76-82. https://doi.org/10.1002/da.10113

Corwith, A., & Ali, F. (2022). The 2020 Pandemic in South Sudan: An Exploration of Teenage Mothers’ and Pregnant Adolescent Girls’ Resilience and Educational Continuity. *Journal on Education in Emergencies, 8*(3), 136-163. https://doi.org/10.33682/81c2-vkk4

Crosnoe, R., & Elder Jr, G. H. (2004). Family dynamics, supportive relationships, and educational resilience during adolescence. *Journal of Family Issues, 25*(5), 571-602. https://doi.org/10.1177/0192513X03258307

Cui, T. X., Kam, C. C. S., Cheng, E. H., & Liu, Q. M. (2022). Exploring the factors relating to academic resilience among students with socioeconomic disadvantages: Factors from individual, school, and family domains. *Psychology in the Schools*. https://doi.org/10.1002/pits.22824

Cunningham, M., & Swanson, D. P. (2010). Educational Resilience in African American Adolescents. *Journal of Negro Education, 79*(4), 473-487.

Cutuli J. J., Herbers J. E., Masten A. S., & Reed, M. G. J. (2016). Resilience in Development. In Snyder C.R., Lopez S. J., Edwards L. M., & Marques S. C. (Eds.), *The Oxford Handbook of Positive Psychology* (3rd Edition). New York, NY.

De Feyter, J. J., Parada, M. D., Hartman, S. C., Curby, T. W., & Winsler, A. (2020). The early academic resilience of children from low-income, immigrant families. *Early Childhood Research Quarterly, 51*, 446-461. https://dx.doi.org/10.1016/j.ecresq.2020.01.001

De Beni, R., Moè, A., Cornoldi, C., Meneghetti, C., Fabris, M., Zamperlin, C., & Tona, GA (2014). Ability and motivation to study: Assessment and orientation tests for secondary school and university. *Erickson Editions: Trento, Italy* .

Dong, Y., & Izadpanah, S. (2022). The effect of corrective feedback from female teachers on formative assessments: educational resilience, educational belongingness, and academic procrastination in an English language course. *Current Psychology,* 1-17. https://doi.org/10.1007/s12144-022-03825-1

Downey, J. A. (2014). Indispensable insight: Children's perspectives on factors and mechanisms that promote educational resilience. *Canadian Journal of Education/Revue canadienne de l'éducation*, *37*(1), 46-71.

Erberer, E., Stephens, M., Mamedova, S., Ferguson, S., & Kroeger, T. (2015). Socioeconomically Disadvantaged Students Who Are Academically Successful: Examining Academic Resilience Cross-Nationally. *IEA Policy Brief series, 5*.

Erlingsson, C., & Brysiewicz, P. (2017). A hands-on guide to doing content analysis. *African Journal of Emergency Medicine, 7*(3), 93-99. https://doi.org/10.1016/j.afjem.2017.08.001

Fang, G., Chan, P. W. K., & Kalogeropoulos, P. (2020). Social support and academic achievement of Chinese low-income children: A mediation effect of academic resilience. *International Journal of Psychological Research, 13*(1), 19-28. https://dx.doi.org/10.21500/20112084.4480

Fantuzzo, J., Leboeuf, W., Rouse, H., Chen, C. C., & Fantuzzo, J. (2012). Academic achievement of African American boys: a city-wide, community-based investigation of risk and resilience. *Journal of School Psychology, 50*(5), 559-579. https://dx.doi.org/10.1016/j.jsp.2012.04.004

Fenwick, A., Kinsella, B., & Harford, J. (2022). Promoting academic resilience in DEIS schools. *Irish Educational Studies*, *41*(3), 513-530. https://doi.org/10.1080/03323315.2022.2094107

Fiorilli, C., Farina, E., Buonomo, I., Costa, S., Romano, L., Larcan, R., & Petrides, K. V. (2020). Trait Emotional Intelligence and School Burnout: The Mediating Role of Resilience and Academic Anxiety in High School. *International Journal of Environmental Research and Public Health, 17*(9). https://doi.org/10.3390/ijerph17093058

Friborg, O., Hjemdal, O., Rosenvinge, J. H., & Martinussen, M. (2003). A new rating scale for adult resilience: what are the central protective resources behind healthy adjustment?. *International Journal of Methods in Psychiatric Research*, *12*(2), 65-76. https://doi.org/10.1002/mpr.143

Gabrielli, G., Longobardi, S., & Strozza, S. (2022). The academic resilience of native and immigrant-origin students in selected European countries. *Journal of Ethnic and Migration Studies, 48*(10), 2347-2368. https://doi.org/10.1080/1369183X.2021.1935657

Garcia-Crespo, F. J., Fernandez-Alonso, R., & Muniz, J. (2021). Academic resilience in European countries: The role of teachers, families, and student profiles. *PLoS ONE, 16*(7). https://dx.doi.org/10.1371/journal.pone.0253409

Garcia-Crespo, F. J., Galian, B., Fernandez-Alonso, R., & Muniz, J. (2019). Educational Resilience in Reading Comprehension: Determinant factors in PIRLS-Europe. *Revista de Educacion*(384), 65-89. https://dx.doi.org/10.4438/1988-592X-RE-2019-384-413

Garcia-Crespo, F. J., Suarez-Alvarez, J., Fernandez-Alonso, R., & Muniz, J. (2022). Academic resilience in mathematics and science: Europe TIMSS-2019 data. *Psicothema, 34*(2), 217-225. <https://doi.org/10.7334/psicothema2021.486>

Gastic, B., & Johnson, D. (2009). Teacher-mentors and the educational resilience of sexual minority youth. *Journal of Gay & Lesbian Social Services*, *21*(2-3), 219-231. https://doi.org/10.1080/10538720902772139

Gayles, J. (2005). Playing the Game and Paying the Price: Academic Resilience among Three High-Achieving African American Males. *Anthropology & Education Quarterly, 36*(3), 250-264. http://dx.doi.org/10.1525/aeq.2005.36.3.250

Ge, T., & Ngai, S. S. Y. (2020). Three pathways to promote poverty resilience: The effects of poverty on children's educational and behavioral performance under multisystems in China. *Children and youth services review, 113*. http://dx.doi.org/10.1016/j.childyouth.2020.104962

Gizir, C. A., & Aydin, G. (2009). Protective Factors Contributing to the Academic Resilience of Students Living in Poverty in Turkey. *Professional School Counseling, 13*(1), 38-49. https://doi.org/10.1177/2156759X0901300103

Gough, D., Oliver, S., & Thomas, J. (2017). *An introduction to systematic reviews* (2nd ed.). SAGE.

Graneheim, U. H., Lindgren, B.-M., & Lundman, B. (2017). Methodological challenges in qualitative content analysis: A discussion paper. *Nurse Education Today*, 56, 29-34. https://doi.org/10.1016/j.nedt.2017.06.002

Granziera, H., Liem, G. A. D., Chong, W. H., Martin, A. J., Collie, R. J., Bishop, M., & Tynan, L. (2022). The role of teachers' instrumental and emotional support in students' academic buoyancy, engagement, and academic skills: A study of high school and elementary school students in different national contexts. *Learning and Instruction, 80*. https://doi.org/10.1016/j.learninstruc.2022.101619

Graves, D. (2014). Black High School Students' Critical Racial Awareness, School-Based Racial Socialization, and Academic Resilience. *Berkeley Review of Education, 5*(1), 5-32. https://doi.org/10.5070/B85110064

Grotberg, E. H. (1995). A guide to promoting resilience in children: strengthening the human spirit. In Early childhood development practice and reflections No. 8 (pp. 5–11). Bernard van Leer Foundation.

Hawkins, R., & Mulkey, L. M. (2005). Athletic Investment and Academic Resilience in a National Sample of African American Females and Males in the Middle Grades. *Education and Urban Society, 38*(1), 62-88. http://dx.doi.org/10.1177/0013124505280025

Hodges, K., & Wong, M. M. (1996). Psychometric characteristics of a multidimensional measure to assess impairment: The Child and Adolescent Functional Assessment Scale. *Journal of Child and Family Studies*, *5*, 445-467. https://doi.org/10.1007/BF02233865

Hofmeyr, H. (2019). *Performance Beyond Expectations: Academic Resilience in South Africa*. Stellenbosch Economic Working Papers: WP19/2019.

Jang, E., Seo, Y. S., & Brutt-Griffler, J. (2023). Building Academic Resilience in Literacy: Digital Reading Practices and Motivational and Cognitive Engagement. *Reading Research Quarterly*. http://dx.doi.org/10.1002/rrq.486

Jaramillo, J., Kothari, B. H., Alley, Z., Rothwell, D., & Blakeslee, J. (2022). Youth-Caseworker Relationship Quality & Academic Resilience Among Transition-Age Youth in Foster Care. *Child and Adolescent Social Work Journal*, 1-16. https://doi.org/10.1007/s10560-022-00906-9

Jin, S. L., Fang, G. B., Cheung, K. C., & Sit, P. S. (2022). Factors associated with academic resilience in disadvantaged students: An analysis based on the PISA 2015 B-S-J-G (China) sample. *Frontiers in Psychology, 13*. https://doi.org/10.3389/fpsyg.2022.84646

Kapikiran, S. (2012). Validity and reliability of the academic resilience scale in Turkish high school. *Education*, *132*(3), 474-484.

Kheirkhah, A. (2020). Investigating the Effect of Social Skills Training on Happiness, Academic Resilience and Self-Efficacy of Girl Students. *Archives of Pharmacy Practice*, *11*(S1), 157-164.

Koirikivi, P., Benjamin, S., Hietajarvi, L., Kuusisto, A., & Gearon, L. (2021). Resourcing resilience: educational considerations for supporting well-being and preventing violent extremism amongst Finnish youth. *International Journal of Adolescence and Youth, 26*(1), 553-569. https://doi.org/10.1080/02673843.2021.2010578

Kim, S. J., Lee, J., Song, J. H., & Lee, Y. (2021). The reciprocal relationship between academic resilience and emotional engagement of students and the effects of participating in the Educational Welfare Priority Support Project in Korea: Autoregressive cross-lagged modeling. *International Journal of Educational Research*, *109*, 101802. https://doi.org/10.1016/j.ijer.2021.101802

Kong, K. (2020). Academic Resilience of Pupils from Low Socioeconomic Backgrounds. *Journal of Behavioral Science, 15*(2), 70-89.

Koirikivi, P., Benjamin, S., Hietajärvi, L., Kuusisto, A., & Gearon, L. (2021). Resourcing resilience: educational considerations for supporting well-being and preventing violent extremism amongst Finnish youth. *International Journal of Adolescence and Youth*, *26*(1), 553-569. <https://doi.org/10.1080/02673843.2021.2010578>

Kosciw, J. G., Palmer, N. A., & Kull, R. M. (2015). Reflecting Resiliency: Openness About Sexual Orientation and/or Gender Identity and Its Relationship to Well-Being and Educational Outcomes for LGBT Students. *American Journal of Community Psychology, 55*(1-2), 167-178. https://doi.org/10.1007/s10464-014-9642-6

Kothari, B. H., Godlewski, B., Lipscomb, S. T., & Jaramillo, J. (2021). Educational resilience among youth in foster care. *Psychology in the Schools, 58*(5), 913-934. https://doi.org/10.1002/pits.22478

Kumi-Yeboah, A. (2020). Educational Resilience and Academic Achievement of Immigrant Students from Ghana in an Urban School Environment. *Urban Education, 55*(5), 753-782. http://dx.doi.org/10.1177/0042085916660347

Kvalsund, R., & Bele, I. V. (2010). Students with special educational needs—Social inclusion or marginalisation? Factors of risk and resilience in the transition between school and early adult life. *Scandinavian Journal of Educational Research*, *54*(1), 15-35. https://doi.org/10.1080/00313830903488445

Langenkamp, A. G. (2010). Academic Vulnerability and Resilience during the Transition to High School: The Role of Social Relationships and District Context. *Sociology of Education, 83*(1), 1-19. http://dx.doi.org/10.1177/0038040709356563

Lei, W., Wang, X., Dai, D. Y., Guo, X., Xiang, S., & Hu, W. (2022). Academic Self-Efficacy and Academic Performance among High School Students: A Moderated Mediation Model of Academic Buoyancy and Social Support. *Psychology in the Schools, 59*(5), 885-899. http://dx.doi.org/10.1002/pits.22653

Lei, W., Zhang, H., Deng, W., Wang, H., Shao, F., & Hu, W. (2021). Academic Self-Efficacy and Test Anxiety in High School Students: A Conditional Process Model of Academic Buoyancy and Peer Support. *School Psychology International, 42*(6), 616-637. http://dx.doi.org/10.1177/01430343211039265

Li, H. (2017). The ‘secrets’ of Chinese students’ academic success: academic resilience among students from highly competitive academic environments. *Educational Psychology*, *37*(8), 1001-1014. https://doi.org/10.1080/01443410.2017.1322179

Li, H., & Yeung W. J. J. (2019). Academic resilience in rural Chinese children: Individual and contextual influences. *Social Indicators Research, 145*(2), 703-717. https://doi.org/10.1007/s11205-017-1757-3

Liebenberg, L., Ungar, M., & LeBlanc, J. C. (2013). The CYRM-12: a brief measure of resilience. *Canadian Journal of Public Health*, *104*, e131-e135. https://doi.org/10.1007/BF03405676

Liew, J., Cao, Q., Hughes, J. N., & Deutz, M. H. (2018). Academic resilience despite early academic adversity: a three-wave longitudinal study on regulation-related resiliency, interpersonal relationships, and achievement in first to third grade. *Early Education and Development, 29*(5), 762-779. https://doi.org/10.1080/10409289.2018.1429766

Liu, B., & Platow, M. J. (2020). Chinese adolescents’ belief in a just world and academic resilience: The mediating role of perceived academic competence. *School Psychology International*, *41*(3), 239-256. https://doi.org/10.1177/0143034320908001

Luthar, S. S., Cicchetti, D., & Becker, B. (2000). The construct of resilience: A critical evaluation and guidelines for future work. *Child development, 71*(3), 543-562. https://doi.org/10.1111/1467-8624.00164

Martin, A. J. (2013). Academic buoyancy and academic resilience: Exploring ‘everyday’ and ‘classic’ resilience in the face of academic adversity. *School Psychology International, 34*(5), 488-500. https://doi.org/10.1177/0143034312472759

Martin, A. (2002). Motivation and academic resilience: Developing a model for student enhancement. *Australian journal of education*, *46*(1), 34-49. https://doi.org/10.1177/000494410204600104

Martin, A. J. (2006). Personal bests (PBs): A proposed multidimensional model and empirical analysis. *British Journal of Educational Psychology*, *76*(4), 803-825. https://doi.org/10.1348/000709905X55389

Martin, A. J., Burns, E. C., Collie, R. J., Cutmore, M., MacLeod, S., & Donlevy, V. (2022). The role of engagement in immigrant students' academic resilience. *Learning and Instruction, 82*. https://doi.org/10.1016/j.learninstruc.2022.101650

Martin, A. J., Colmar, S. H., Davey, L. A., & Marsh, H. W. (2010). Longitudinal modelling of academic buoyancy and motivation: Do the 5Cs hold up over time? *British Journal of Educational Psychology, 80*(3), 473-496. https://doi.org/10.1348/000709910X486376

Martin, A. J., Ginns, P., Brackett, M. A., Malmberg, L. E., & Hall, J. (2013). Academic buoyancy and psychological risk: Exploring reciprocal relationships. *Learning and Individual Differences, 27*, 128-133. https://dx.doi.org/10.1016/j.lindif.2013.06.006

Martin, A. J., & Marsh, H. W. (2008). Academic buoyancy: Towards an understanding of students' everyday academic resilience. *Journal of school psychology, 46*(1), 53-83. https://dx.doi.org/10.1016/j.jsp.2007.01.002

Masten, A. S. (2001). Ordinary magic: Resilience processes in development. *American Psychologist, 56*, 227–238. https://doi.org/10.1037/0003-066X.56.3.227

McGrath, T. A., Alabousi, M., Skidmore, B., Korevaar, D. A., Bossuyt, P. M., Moher, D., ... & McInnes, M. D. (2017). Recommendations for reporting of systematic reviews and meta-analyses of diagnostic test accuracy: a systematic review. *Systematic Reviews*, *6*(1), 1-15. https://doi.org/10.1186/s13643-017-0590-8

Miller, S., Connolly, P., & Maguire, L. K. (2013). Wellbeing, academic buoyancy and educational achievement in primary school students. *International Journal of Educational Research*, *62*, 239-248. https://doi.org/10.1016/j.ijer.2013.05.004

Mohan, V., & Kaur, J. (2021). Assessing the relationship between grit and academic resilience among students. *Issues and Ideas in Education, 9*(1), 39-47.

Morales, E. E. (2008). Academic resilience in retrospect: Following up a decade later. *Journal of Hispanic Higher Education, 7*(3), 228-248. https://doi.org/10.1177/1538192708317119

Mullis, I. V. S., & Martin, M. O. (2017). *TIMSS 2019 Assessment Frameworks*. Retrieved from Boston College, TIMSS & PIRLS International Study Center website: <http://timssandpirls.bc.edu/timss2019/frameworks/>.

Neal, D. (2017). Academic resilience and caring adults: The experiences of former foster youth. *Children and Youth Services Review, 79*, 242-248. <https://dx.doi.org/10.1016/j.childyouth.2017.06.005>

Nichols, E. B., Loper, A. B., & Meyer, J. P. (2016). Promoting educational resiliency in youth with incarcerated parents: The impact of parental incarceration, school characteristics, and connectedness on school outcomes. *Journal of Youth and Adolescence, 45*(6), 1090-1109. <https://dx.doi.org/10.1007/s10964-015-0337-6>

Nota, L., Soresi, S., & Zimmerman, B. J. (2004). Self-Regulation and Academic Achievement and Resilience: A Longitudinal Study. *International Journal of Educational Research, 41*(3), 198-215. http://dx.doi.org/10.1016/j.ijer.2005.07.001

Oldfield, J., Stevenson, A., & Ortiz, E. (2020). Promoting resilience in street connected young people in Guatemala: The role of psychological and educational protective factors. *Journal of community psychology, 48*(2), 590-604. https://doi.org/10.1002/jcop.22272

Özcan, B., & Bulus, M. (2022). Protective factors associated with academic resilience of adolescents in individualist and collectivist cultures: Evidence from PISA 2018 large scale assessment. *Current Psychology, 41*(4), 1740-1756. https://doi.org/10.1007/s12144-022-02944-z

Paat, Y. F. (2015). Children of Mexican Immigrants' Aspiration-Attainment Gap and Educational Resilience. *Diaspora, Indigenous, and Minority Education, 9*(1), 37-53. http://dx.doi.org/10.1080/15595692.2014.980804

Pan, E. L., & Yi, C. C. (2011). Constructing educational resilience: The developmental trajectory of vulnerable Taiwanese youth. *Journal of Comparative Family Studies,* *42*(3), 369-384. https://doi.org/10.3138/jcfs.42.3.369

Peck, S. C., Roeser, R. W., Zarrett, N., & Eccles, J. S. (2008). Exploring the roles of extracurricular activity quantity and quality in the educational resilience of vulnerable adolescents: Variable- and pattern-centered approaches. *Journal of Social Issues, 64*(1), 135-155. https://doi.org/10.1111/j.1540-4560.2008.00552.x

Peck, S. C., Roeser, R. W., Zarrett, N., & Eccles, J. S. (2008). "Exploring the roles of extracurricular activity quantity and quality in the educational resilience of vulnerable adolescents: Variable- and pattern-centered approaches": Erratum. *Journal of Social Issues, 64*(2), 430. https://doi.org/10.1111/j.1540-4560.2008.00552.x

Plunkett, S. W., Henry, C. S., Houltberg, B. J., Sands, T., & Abarca-Mortensen, S. (2008). Academic support by significant others and educational resilience in Mexican-origin ninth grade students from intact families. *The Journal of Early Adolescence*, *28*(3), 333-355. https://doi.org/10.1177/0272431608314660

Putwain, D. W., & Daly, A. L. (2013). Do clusters of test anxiety and academic buoyancy differentially predict academic performance? *Learning and Individual Differences, 27*, 157-162. https://doi.org/10.1016/j.lindif.2013.07.010

Rachmawati, I., Setyosari, P., Handarini, D. M., & Hambali, I. (2021). Do social support and self-efficacy correlate with academic resilience among adolescence. *International Journal of Learning and Change, 13*(1), 49-62. https://doi.org/10.1504/IJLC.2021.111664

Rana, M., Qin, D. B., Bates, L., Luster, T., & Saltarelli, A. (2011). Factors related to educational resilience among Sudanese unaccompanied minors. *Teachers College Record, 113*(9), 2080-2114. https://doi.org/10.1177/016146811111300905

Randolph, K. A., Fraser, M. W., & Orthner, D. K. (2004). Educational Resilience Among Youth at Risk. *Substance use & misuse, 39*(5), 747-767. <https://dx.doi.org/10.1081/JA-120034014>

Ricketts, S. N., Engelhard Jr, G., & Chang, M. L. (2015). Development and validation of a scale to measure academic resilience in mathematics. *European Journal of Psychological Assessment*. https://doi.org/10.1027/1015-5759/a000274

Rivera, H., Waxman, H. C., & Powers, R. (2012). English Language Learners' Educational Resilience and Classroom Learning Environment. *Educational Research Quarterly*, *35*(4), 57-78.

Rojas Flórez, L. F. (2015). Factors Affecting Academic Resilience in Middle School Students: A Case Study (Factores que Afectan la Resiliencia Académica en Estudiantes de Bachillerato). *GIST Education and Learning Research Journal*(11), 63-78.

Rosen, J. A., Warkentien, S., & Rotermund, S. (2019). Stopping out versus Dropping Out: The Role of Educational Resilience in Explaining On-Time Completion of High School. *American Journal of Education, 125*(2), 259-287. https://doi.org/10.1086/701248

Rudd, G., Meissel, K., & Meyer, F. (2021). Measuring academic resilience in quantitative research: A systematic review of the literature. *Educational Research Review, 34*, 100402. https://doi.org/10.1016/j.edurev.2021.100402

Rutkowski, L., Gonzalez, E., Joncas, M., & Von Davier, M. (2010). International Large-Scale Assessment Data: Issues in Secondary Analysis and Reporting. *Educational Researcher, 39*(2), 142-151. https://doi.org/10.3102/0013189X10363170

Sacker, A., & Schoon, I. (2007). Educational resilience in later life: Resources and assets in adolescence and return to education after leaving school at age 16. *Social Science Research, 36*(3), 873-896. https://dx.doi.org/10.1016/j.ssresearch.2006.06.002

Salvo-Garrido, S., Vargas, H. M., Urra, O. V., Gálvez-Nieto, J. L., & Miranda-Zapata, E. (2019). Students with socioeconomic disadvantages who have academic success in language: Examining academic resilience in South America. *Preprints.org,* 2019120043. https://doi.org/10.20944/preprints201912.0043.v1

Samuels, W. E. (2004). *Development of a non-intellective measure of academic success: Towards the quantification of resilience*. The University of Texas at Arlington.

Sandoval-Hernández, A., & Bialowolski, P. (2016). Factors and Conditions Promoting Academic Resilience: A TIMSS-Based Analysis of Five Asian Education Systems. *Asia Pacific Education Review, 17*(3), 511-520. https://doi.org/10.1007/s12564-016-9447-4

Sandoval-Hernandez, A., & Biaowolski, P. (2016). Factors and conditions promoting academic resilience: a TIMSS-based analysis of five Asian education systems. *Asia Pacific Education Review, 17*(3), 511-520. https://doi.org/10.1007/s12564-016-9447-4

Schelble, J. L., Franks, B. A., & Miller, M. D. (2010). Emotion Dysregulation and Academic Resilience in Maltreated Children. *Child & Youth Care Forum, 39*(4), 289-303. http://dx.doi.org/10.1007/s10566-010-9105-7

Schoon, I., Parsons, S., & Sacker, A. (2004). Socioeconomic Adversity, Educational Resilience, and Subsequent Levels of Adult Adaptation. *Journal of Adolescent Research, 19*(4), 383-404. http://dx.doi.org/10.1177/0743558403258856

Shao, Y. H., & Kang, S. M. (2022). The association between peer relationship and learning engagement among adolescents: The chain mediating roles of self-efficacy and academic resilience. *Frontiers in Psychology, 13*. https://doi.org/10.3389/fpsyg.2022.938756

Skinner, E. A., & Pitzer, J. R. (2012). Developmental dynamics of engagement, coping, and everyday resilience. In S. Christenson, A. Reschly, & C. Wylie (Eds.), *The handbook of research on student engagement* (pp. 21-44). New York, NY: Springer Science.

Skinner, E., Pitzer, J., & Steele, J. (2013). Coping as part of motivational resilience in school: A multidimensional measure of families, allocations, and profiles of academic coping. *Educational and Psychological Measurement*, *73*(5), 803-835. https://doi.org/10.1177/0013164413485241

Stevenson, A. D., Gallard Martínez, A. J., Brkich, K. L., Flores, B. B., Claeys, L., & Pitts, W. (2019). Latinas’ heritage language as a source of resiliency: Impact on academic achievement in STEM fields. *Cultural Studies of Science Education*, *14*, 1-13. https://doi.org/10.1007/s11422-016-9789-6

Strolin-Goltzman, J., Woodhouse, V., Suter, J., & Werrbach, M. (2016). A mixed method study on educational well-being and resilience among youth in foster care. *Children and Youth Services Review, 70*, 30-36. https://doi.org/10.1016/j.childyouth.2016.08.014

Süleyman, A. (2022). Investigation of the Individual Characteristics that Predict Academic Resilience. *International Journal of Contemporary Educational Research, 9*(3), 543-556. https://doi.org/10.33200/ijcer.1076091

Thiessen, V. (2008). Resilience and educational pathways: A longitudinal analysis of low reading achievers. *Canadian Journal of Family and Youth/Le Journal Canadien de Famille et de la Jeunesse*, 27-62. https://doi.org/10.29173/cjfy6047

Tinsley, B., & Spencer, M. B. (2010). High hope and low regard: The resiliency of adolescents' educational expectations while developing in challenging political contexts. *Research in Human Development*, *7*(3), 183-201. https://doi.org/10.1080/15427609.2010.505780

Trigueros, R., Aguilar-Parra, J. M., Cangas, A. J., Bermejo, R., Ferrandiz, C., & López-Liria, R. (2019). Influence of emotional intelligence, motivation and resilience on academic performance and the adoption of healthy lifestyle habits among adolescents. *International Journal of Environmental Research and Public Health*, *16*(16), 2810. https://doi.org/10.3390/ijerph16162810

Trigueros, R., Lirola, M. J., Cangas, A. J., Aguilar-Parra, J. M., García-Mas, A., & Trigueros, R. (2022). Is resilience learned through the frustration of the BPN? An empirical study about its role in the acquisition of positive lifestyles and academic outcomes framed in SDT. *Current Psychology*, 1-10. https://doi.org/10.1007/s12144-022-03496-y

Tudor, K. E., & Spray, C. M. (2017). Approaches to measuring academic resilience: A systematic review. *International Journal of Research Studies in Education, 7*(4). https://doi.org/10.5861/ijrse.2017.1880

Ungar, M., & Liebenberg, L. (2011). Assessing Resilience Across Cultures Using Mixed Methods: Construction of the Childand Youth Resilience Measure. *Journal of Mixed Methods Research, 5*(2), 126–149. https://doi.org/10.1177/1558689811400607

Vicente, I., Pastor, J. M., & Soler, A. (2021). Improving educational resilience in the OECD countries: Two convergent paths. *Journal of Policy Modeling, 43*(6), 1149-1166. https://doi.org/10.1016/j.jpolmod.2021.09.007

Victor-Aigboidion, V., Onyishi, C. N., & Ngwoke, D. U. (2020). Predictive power of academic self-efficacy on academic resilience among secondary school students. *Journal of the Nigerian Council of Educational Psychologists, 12*(1).

Watson, L. L., & Vogel, L. R. (2017). Educational Resiliency in Teen Mothers. *Cogent Education, 4*(1). http://dx.doi.org/10.1080/2331186X.2016.1276009

Wayman, J. C. (2002). The utility of educational resilience for studying degree attainment in school dropouts. *The Journal of Educational Research, 95*(3), 167-178. https://doi.org/10.1080/00220670209596587

Werner, E. E. (2000). Protective factors and individual resilience. *Handbook of early childhood intervention, 2*, 115-132.

Williams, K. E., Berthelsen, D., & Laurens, K. R. (2022). Academic resilience from school entry to third grade: Child, parenting, and school factors associated with closing competency gaps. *Plos one*, *17*(11), e0277551. https://doi.org/10.1371/journal.pone.0277551

Wills, G., & Hofmeyr, H. (2019). Academic resilience in challenging contexts: Evidence from township and rural primary schools in South Africa. *International Journal of Educational Research, 98*, 192-205. https://doi.org/10.1016/j.ijer.2019.08.001

Wittrup, A. R., Hussain, S. B., Albright, J. N., Hurd, N. M., Varner, F. A., & Mattis, J. S. (2019). Natural mentors, racial pride, and academic engagement among black adolescents: Resilience in the context of perceived discrimination. *Youth & Society*, *51*(4), 463-483. https://doi.org/10.1177/0044118X16680546

Wolke, D., Jaekel, J., Hall, J., & Baumann, N. (2013). Effects of sensitive parenting on the academic resilience of very preterm and very low birth weight adolescents. *Journal of Adolescent Health, 53*(5), 642-647. https://dx.doi.org/10.1016/j.jadohealth.2013.06.014

Wood, M., Liebenberg, L., Ikeda, J., & Vincent, A. (2020). The Role of Educational Spaces in Supporting Inuit Youth Resilience. *Child Care in Practice, 26*(4), 390-415. https://doi.org/10.1080/13575279.2020.1765143

Wu, Q., Tsang, B., & Ming, H. (2014). Social capital, family support, resilience and educational outcomes of Chinese migrant children. *British Journal of Social Work*, *44*(3), 636-656. https://doi.org/10.1093/bjsw/bcs139

Yavuz, H. C., & Kutlu, O. (2016). Investigation of the Factors Affecting the Academic Resilience of Economically Disadvantaged High School Students. *EGITIM VE BILIM-EDUCATION AND SCIENCE, 41*(186), 1-19. https://doi.org/10.15390/EB.2016.5497

Yu, K., & Martin, A. J. (2014). Personal Best (PB) and "Classic" Achievement Goals in the Chinese Context: Their Role in Predicting Academic Motivation, Engagement and Buoyancy. *Educational Psychology, 34*(5), 635-658. http://dx.doi.org/10.1080/01443410.2014.895297

Yu, K., Martin, A. J., Hou, Y., Osborn, J., & Zhan, X. (2019). Motivation, engagement, academic buoyancy, and adaptability: The roles of socio-demographics among middle school students in China. *Measurement: Interdisciplinary Research and Perspectives*, *17*(3), 119-132. https://doi.org/10.1080/15366367.2019.1584518

Yustika, Y. & Widyasari, P. (2021). Suryaratri, R.D., Zakiah, E., & Oktoriva, A.E. (2021). Students’self-compassion and academic resilience in pandemic era. *International Journal of Research in Counseling and Education, 5*(2), 195-205, https://doi.org/10.24036/00458za0002

Zaw, A. M. M., Win, N. Z., & Thepthien, B. O. (2022). Adolescents' academic achievement, mental health, and adverse behaviors: Understanding the role of resilience and adverse childhood experiences. *School Psychology International, 43*(5), 516-536. https://doi.org/10.1177/01430343221107114
